# Clinicopathological and Radiological Features of Cats Presented with Infectious Respiratory Disease Signs: A Focus on *Rhodococcus equi* and *Klebsiella pneumoniae*

**DOI:** 10.3390/microorganisms11030737

**Published:** 2023-03-14

**Authors:** Muhammad Waseem Aslam, Seng Fong Lau, Rozanaliza Radzi, Sharina Omar, Ubedullah Kaka, Ishtiaq Ahmed

**Affiliations:** 1 Department of Veterinary Clinical Studies, Faculty of Veterinary Medicine, University Putra Malaysia, Serdang 43400, Malaysia; 2 Department of Veterinary Pathology and Microbiology, Faculty of Veterinary Medicine, University Putra Malaysia, Serdang 43400, Malaysia; 3 Department of Pathobiology, University of Veterinary and Animal Sciences Lahore, Sub-Campus Jhang, Jhang, Pakistan

**Keywords:** *Rhodococcus equi*, *Klebsiella pneumoniae*, cat, PCR, histopathology, URT, LRT

## Abstract

The objective of this study was to evaluate the prevalence of involvement of common viral organisms *R. equi* and *K. pneumoniae* and their clinicopathological and radiological features in respiratory disease of Malaysian domestic cats. A total of 34 feline cases with acute/chronic infectious respiratory disease signs were followed prospectively to investigate respiratory disease due to *R. equi* and *K. pneumoniae* and their relationship with concurrent viral infections in disease manifestation. All sampled cats (*n* = 27) were positive for FCoV antibodies and negative for FeLV. A significantly high antibody titer for FCV in *n* = 26 cases was also noticed. A single sample of pyothorax from a 3-months-old, non-vaccinated kitten was positive for *R. equi*. Bronchopneumonia with severe infiltration of the polymorphs and mononuclear inflammatory cells were prominent features of lungs histopathology from the kitten positive for *R. equi*. *K. pneumoniae* subsp. *pneumoniae* was confirmed from tracheal swabs of two cats. Histologically, the tracheal tissues of the two cats positive for *K. pneumoniae* were normal. In diagnostic imaging, epicenter of the infectious URT disease was nasal conchae rostrally and nasal turbinates caudally, however for infectious LRT disease was bronchial tree. Conclusively, infectious respiratory disease is a complex illness in cats, predominantly for unvaccinated kittens and young adult cats, especially those kept in multi-cat household or shelter environments because of the involvement of multiple bacterial and viral organisms as primary or secondary invaders. Clinicians should not preclude feline rhodococcosis from differentials, especially in kittens with pyothorax and less than one year of age. Unlike *R. equi*, *K. pneumoniae* has the potential to colonize URT of cats which might be disseminating further to cause LRT disease.

## 1. Introduction

In a variety of disorders affecting upper respiratory tract (URT) of cats, infectious upper respiratory diseases caused by viral pathogens are by far the most common one. Feline herpes virus type 1 (FHV-1) and feline calicivirus (FCV) account approximately 90% in feline URT infections [1]. Similarly, a number of bacteria, parasites, protozoa, fungi and viruses have been reported in lower respiratory tract (LRT) diseases of the cats [2]. The reports are emerging about the presence of multi-drug resistance *Rhodococcus equi* (*R. equi*) and *Klebsiella pneumoniae* (*K. pneumoniae*) in Malaysian environment [3,4,5]. Upper and/or lower respiratory infections from these bacteria are quite uncommon in this specie and reported in past [3,6,7].

From 2012 until 2018, a significant rise in the respiratory disease due to the multi-drug resistant *R. equi* has been reported in Malaysian domestic cats. There are very few reports on feline rhodococcosis particularly for pulmonary form of the disease [8,9,10]. Contrary to the impression given by previous reports on feline rhodococcosis, pulmonary form of the disease have been reported more commonly (36/40) compared with cutaneous form of the disease (4/40) in Malaysian domestic cats [3]. To the best of author’s knowledge colonization and respiratory disease due to *K. pneumoniae* has never been reported in Malaysian domestic cats. In present study, the focus was more towards respiratory infections of cats with Gram-positive *R. equi* and Gram-negative *K. pneumoniae* bacteria. The objectives of this study were to investigate cats with infectious respiratory disease signs, and report their clinicopathological, radiographic, and computed tomographic findings especially related to the focused bacteria and correlate the disease with concurrent viral infections, where indicated.

## 2. Materials and Methods

Ethical approval for this study was obtained from Institutional Animal Care and Use Committee of Universiti Putra Malaysia (AUP-R037/2019). Cats were recruited from shelters and private clinics based on the criteria of acute or chronic severe infectious upper/lower/mixed respiratory disease signs. Furthermore, all privately owned cats were diagnosed with pyothorax. The selected population of the shelter cats was already isolated and being subjected to euthanasia due to over-population. After physical examination, all shelter cats were anaesthetized by 5mg/kg of Zoletil 100 (Virbac, Carros, France) administered intramuscularly for further sampling.

### 2.1. Sampling

A total of 3–5 mL of blood was collected from the jugular vein of each cat (shelter and privately owned) in K_3_ EDTA and plain tubes and centrifuged at 4000 rpm for 5–10 min. Serum and plasma samples were stored at –80 °C for further study. Besides hematology and serum biochemistry, all blood samples were analyzed for qualitative FeLV antigen, FIV and FCoV antibody snap tests (Bionote Inc., Hwaseong, Republic of Korea), and semi-quantitative FCV and FHV IgG immunoassay tested by Vcheck-V200 machine (Bionote Inc., Republic of Korea).

In pyothorax cases, pleural effusion and sterile cotton swabs of nasal discharge were submitted for bacterial culture and susceptibility. While in complicated cases where owners opted euthanasia, post-mortem was performed. During the post-mortem valuation was performed: sampling of the trachea and lung tissues along with sterile cotton swabs of the cranial trachea and lungs were collected aseptically for each cat for bacterial culture, tissue samples for histopathology. Trachea and whole lungs were extracted and examined by the methodology adapted from Falsone et al. [11] for the presence of lungworm(s). Furthermore during necropsy, stool samples were also collected from distal colon and subjected to faecal Baermann test as described by the Taubert et al. [12] to detect the presence of lungworm(s) larvae in stool samples.

### 2.2. Bacterial Culture and Confirmation

Samples were cultured at 37 °C with an incubation period of 48–72 h on 5% defibrinated horse blood agar and MacConkey agar for the detection of *R. equi* and *K. pneumoniae* growth. All collected swab samples and isolated colonies of *R. equi* and *K. pneumoniae* on growth media (confirmed by conventional biochemical testing) were processed further with polymerase chain reaction (PCR) for the detection (in case desired bacterial colony was missed in primary colony growth) and confirmation of these bacteria where growth indicated. Reference strains *R. equi* ATCC 6939 and *K. pneumoniae* subsp. *pneumoniae* ATCC 13883 were used as positive control.

DNA from all these samples and positive control were extracted using a quick-start protocol of DNeasy^®^ Blood and Tissue Kit (Qiagen, Hilden, Germany). Although, this extraction kit is not holding any standard protocol for DNA extraction from swab samples and/or freshly cultured bacterial colonies, but a cultured cells protocol was modified successfully to extract bacterial genomic DNA from tested samples and control. Initially, sampled swab was placed in 1.5 mL tube for each sample and resuspended in 200 µL of phosphate buffered saline (PBS), then 20 µL of proteinase K was added in each tube. In the next step, a 200µL of buffer AL was added in each tube and mixed thoroughly by vortexing. Then, samples were Incubated at 56 °C for 10 min and the swabs were removed from the tube before proceeding any further steps. Rest of the DNeasy spin column, centrifugation and elution steps were followed according to the manufacturer’s recommended protocol.

DNA was subjected to PCR using two sets of primers; targeting specific partial sequence of 16S rDNA and *choE* genes for *K. pneumoniae* and *R. equi* respectively, as shown in Table 1. PCR protocols were optimized with “SensoQuest Labcycler, Germany” for both bacteria before proceeding with actual samples. A slight modification was needed for PCR reaction and gel electrophoresis (Table 2) compared to the previously described protocols by Tayebeh et al. [13] and Ladrón et al. [14] for a similar sequence of primers for the detection and/or confirmation of *K. pneumoniae* and *R. equi* respectively. RNase-free water was used as a negative control in all PCR reactions. The gel for electrophoresis was stained with SYBRSafe DNA Gel stain (Invitrogen, Carlsbad, CA, EUA). The stained amplicons were captured by the alpha imager 2200 software with GelDoc (Alphalmager™, San Leandro, CA, USA).

### 2.3. Diagnostic Imaging

In diagnostic imaging, either thoracic radiographic (left, right lateral and dorsoventral) and/or computed tomography scan (CT scan) of the head, neck and thorax until diaphragm were performed with the owner’s consent. Radiographs and CT scans were reviewed by two veterinarians to reach a consensus. Severity and interpretive criteria were adapted from previous studies [3,15,16]. The severity criteria for CT scan findings were categorized as “mild” when pathological changes were affecting ≤25% of any involved structure, “moderate” when pathology was affecting 25–50% of any involved structure and “severe” when pathology was affecting majority of the involved structure. To summarize results and encompass maximum possible pathologies, CT scan findings were studied around six regions of the upper and lower respiratory tract: (1) nasal vestibule region, (2) maxilloturbinate region, (3) ethmoidal region, (4) frontal sinus region, (5) neck region and (6) thoracic region. Additionally, regional lymph nodes (mandibular, parotid, retropharyngeal, tracheobronchial and retrosternal), bilateral ear canal, bilateral fascial planes of skull, neck and thoracic cavity were also examined when abnormalities were related to upper or lower respiratory system.

### 2.4. Histopathology

Histology and H&E staining of the trachea and lungs were performed on formalin fixed tissues. Aseptically collected sample tissues were adequate in size and covering portions of the gross lesion(s) and normal tissue in a single section (where available). For further processing, fixation was done in 10% buffered formalin, trimming was done through tissue trimming blades, and sectioning (4 μm) of the embedded paraffin blocks was done through microtome. Finally, H&E staining was done, and slides were examined under a light microscope attached with a camera source (Leica DFC 295, Wetzlar, Germany). Cellular changes such as edema, hemorrhage and congestion, inflammatory cells infiltration, necrosis and degeneration were recorded for each sample. The cellular changes were scored into 4 categories included: normal (normal tissue); mild (≤25% tissue affected); moderate (≤50% tissue affected); and severe (>50% tissue affected).

## 3. Results

### 3.1. Signalment and History

From February 2019 until November 2019, 34 DSH cats were recruited for a prospective case study. Twenty-two cats with acute/chronic infectious respiratory disease signs (10 females and 12 males; unknown age) were recruited from two shelters. Another twelve cats diagnosed with pyothorax (5 females and 7 males; 3 months to <2 years) were privately owned cats.

### 3.2. Clinical Findings

The mean body temperature for shelter cats was 38.9 ± 0.17 °C (*n* = 22). Mean body temperature could not be retrieved for privately owned cats. Upon clinical examination of shelter cats, pronounced respiratory signs were stertorous breathing (63%), sneezing and serous nasal discharge (55%), mucoid nasal discharge (45%), unilateral/bilateral epiphora (50%), encrustation of the eyelids and external nares (36%). However, concurrent non-specific signs such as lethargy (77%), low body condition score (73%) and dehydration (63%) were predominant findings in shelter cats. Oral ulceration, breathing abnormalities, conjunctivitis, coughing and mandibular lymphadenomegaly were also noticed in few shelter cats, as shown in Table 3. Respiratory distress with abnormal respiratory sounds and non-specific signs such as anorexia/hyporexia were seen in all privately owned cats diagnosed with pyothorax (Table 3).

### 3.3. Hematological and Serum Biochemistry Results

**Figure 1:**
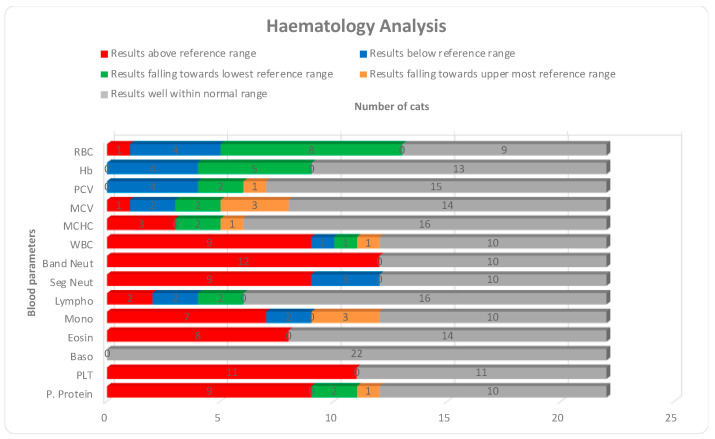
Comparison of changes in hematology parameters in a total of 22 cats with reference to their reference range.

**Figure 2:**
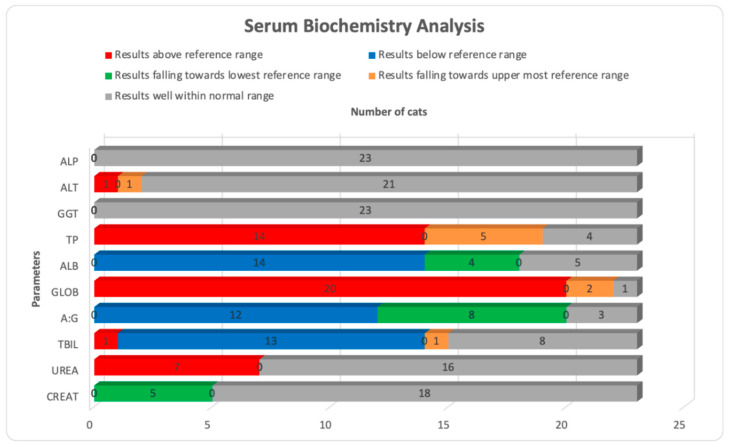
Comparison of changes in serum biochemistry parameters in a total of 23 cats with reference to their reference range.

Hematological and serum biochemistry results are presented in Table 4 and Table 5. Of the 22 available hematological results, the most significant abnormalities were leukocytosis with band-neutrophilia, segmented neutrophilia, monocytosis, eosinophilia and elevated plasma proteins noticed in 41% (*n* = 9), 55% (*n* = 12), 41% (*n* = 9), 32% (*n* = 7), 36% (*n* = 8), and 41% (*n* = 9) samples, respectively. Thrombocytopenia was confirmed with blood smears and noticed in 50% (*n* = 11) of the samples. Changes such as lymphopenia, low red blood cells, low hemoglobin concentration and low packed cell volume were also noticed in few samples, as shown in Figure 1. Of the 23 available serum biochemistry results, changes in protein levels were the most significant findings. Hypoalbuminemia with hyperglobulinemia which also altered the albumin to globulin (A:G) ratio was noticed in 61% (*n* = 14) and 87% (*n* = 20) samples, respectively. A low A:G ratio was calculated in 52% (*n* = 12) samples. A significant number of samples had similar changes in proteins and related parameters when their results were analyzed for upper most and lowest reference limits, as shown in Figure 2. An A:G ratio was recorded at the lowest reference range in 35% (*n* = 8) of the samples. Only urea was elevated in 30% (*n* = 7) samples, while creatinine was recorded on the lowest reference range in 22% (*n* = 5) of the samples. Alkaline phosphatase and gamma-glutamyl transferase were well within reference range in all 23 samples.

### 3.4. Lungworm and Viral Screening Results

**Figure 3:**
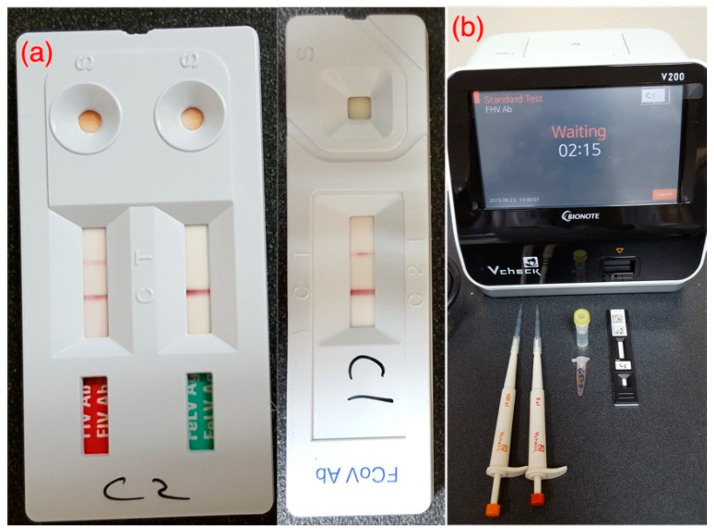
(**a**) Qualitative snap tests and (**b**) Vcheck-V200 machine for semi-quantitative IgG immunoassays.

Fecal Baermann test and post-mortem gross and microscopic examination of the trachea and lungs were negative for all examined cats (*n* = 23). Viral screening through qualitative snap tests [Figure 3a] and semi-quantitative IgG immunoassays through Vcheck-V200 machine [Figure 3b] have been outlined in Table 6. In qualitative snap tests, 100% (*n* = 27) samples were positive for FCoV antibodies, and 15% (*n* = 4) of samples were positive for FIV, while only one cat (4%) was positive for FeLV. For semi-quantitative IgG immunoassays, by default, the antibody titer is scaled from (1) to (6) by the machine to describe the virus neutralization at a specific dilution. Scale (1) to (2) is categorized as “low titer”. Scale (3) is “medium” while scale (4) until (6) is categorized as “high” antibody titer. Overall, 96% (*n* = 26) samples produced high antibody titer [scale (4) to (6)] for FCV, while highest scale (6) was recorded in 78% (*n* = 21) samples. Similarly, a high antibody titer [scale (4) to (6)] for FHV was recorded in 30% (*n* = 8) samples, while low and medium titers were recorded in 26% (*n* = 7) and 22% (*n* = 6) samples, respectively.

### 3.5. Bacterial Culture and Confirmation Results

Table 7 indicates the complete sequel of individual sample collected from all 34 cats with final outcome. Tracheal and nasal swabs were collected from shelters’ cats while only nasal swabs from privately-owned cats for URT infection(s). Similarly, lung swabs were collected from shelters’ cats while pleural effusions from privately-owned cats. A total of 62% (*n* = 21) samples from URT and 38% (*n* = 13) samples from LRT produced some kind of bacterial growth(s) on blood agar. Same swabs were used on MacConkey agar and produced some growth(s) from 24% (*n* = 8) samples collected from URT and 9% (*n* = 3) samples from LRT.

**Figure 4:**
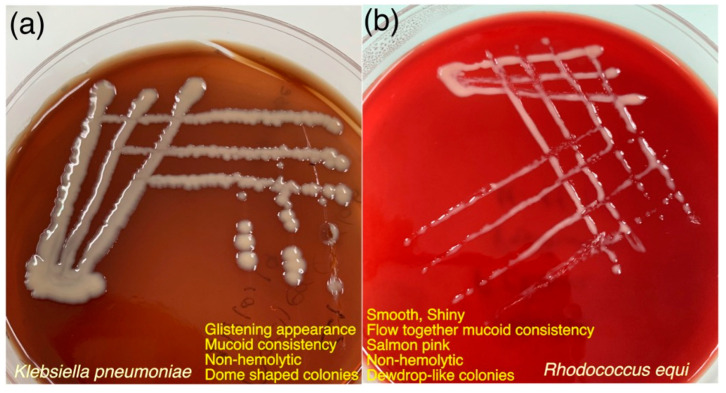
Growth appearance and colony characteristics of (**a**) *K. pneumoniae* and (**b**) *R. equi* on 5% defibrinated horse blood agar, isolated from trachea and pleural effusion samples of two cats investigated for acute/chronic infectious respiratory disease signs.

**Figure 5:**
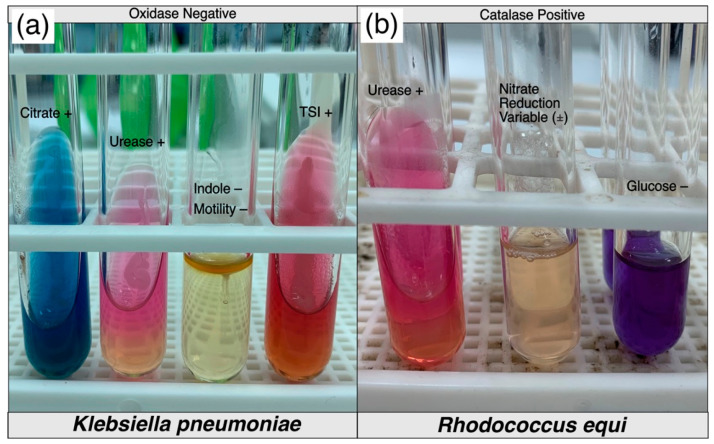
Conventional biochemical testing results of (**a**) *K. pneumoniae* and (**b**) *R. equi*, isolated from trachea and pleural effusion samples of two cats investigated for acute/chronic infectious respiratory disease signs.

**Figure 6:**
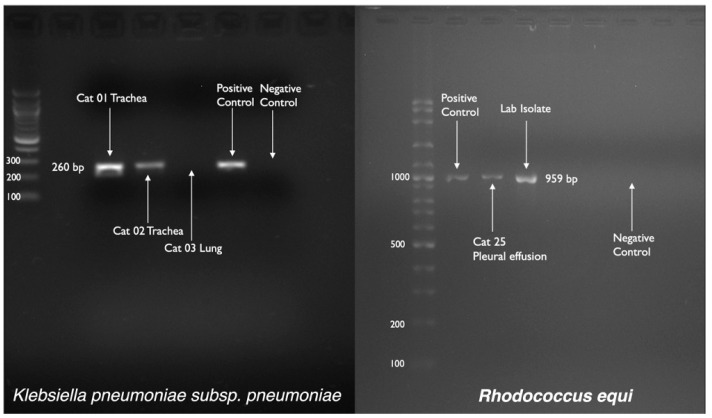
PCR confirmation results of the biochemically confirmed bacterial colonies.

**Figure 7:**
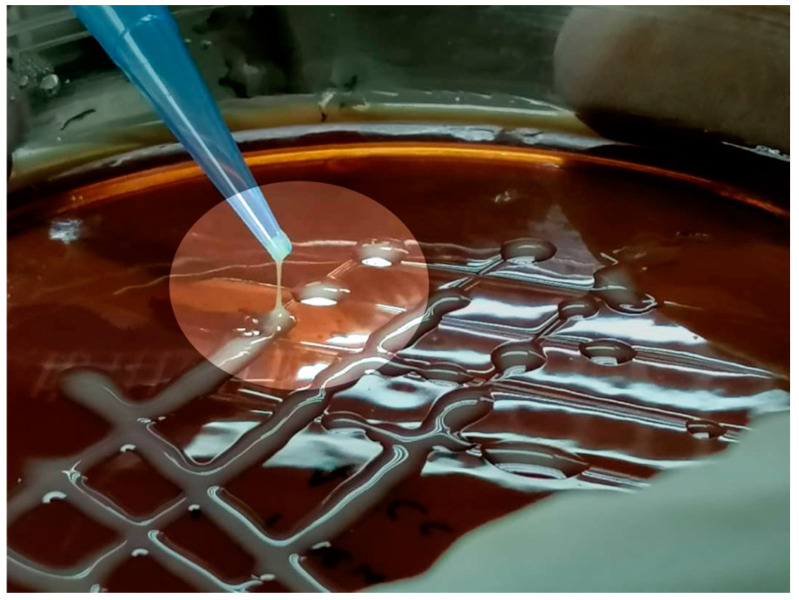
“String test” indicating hypermucoviscous colony of *Klebsiella pneumoniae* from tracheal swab of Cat 02. A > 5 mm viscous string is the indicator of positive result.

Suspected samples with colony characteristics of *K. pneumoniae* and *R. equi,* as shown in Figure 4a,b, were processed further with conventional biochemical testing. A summary of the conventional biochemical testing for processed samples has been outlined in Table 8 and biochemical changes in each test for both bacteria have been shown in Figure 5a,b. Tracheal swabs from Cat 01 and 02 were specifically positive for *K. pneumoniae* while another lung swab from Cat 03 was positive for an unidentified *Klebsiella* spp. which was finally negative for *K. pneumoniae* subsp. *pneumoniae* on further confirmation with conventional PCR (Figure 6). *R. equi* was isolated from a single pleural effusion sample of Cat 25 [Figure 4b] and confirmed with both biochemical [Figure 5b] and molecular technique, PCR (Figure 6). Tracheal swab of the Cat 02 produced a hypermucoviscous colony, as shown in Figure 7.

### 3.6. Radiological Findings

**Figure 8:**
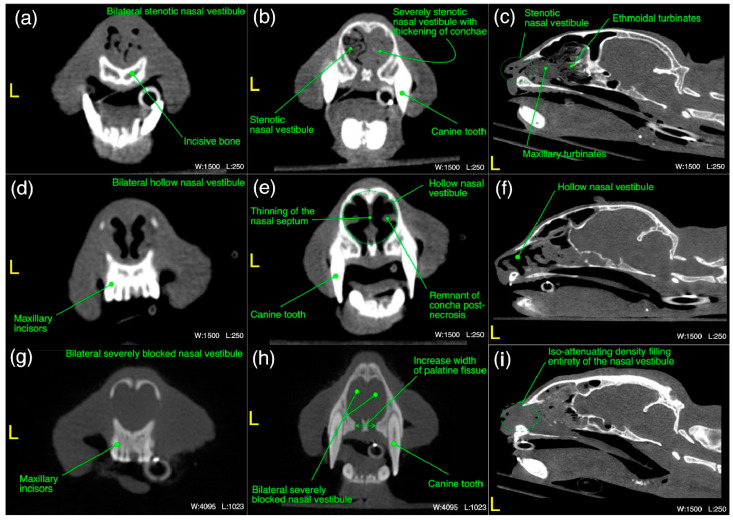
(**a**–**i**) A variety of computed tomographic changes of nasal vestibule region are labeled for pathologies related to acute/chronic infectious respiratory disease signs in Cats 1, 10 and 20. Window, level and laterality are also labeled for each section.

**Figure 9:**
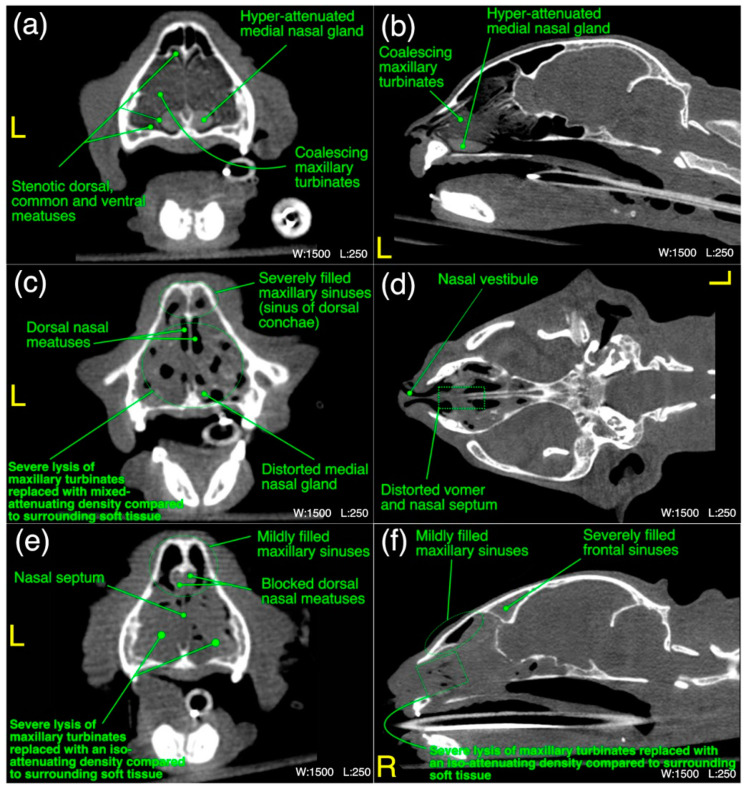
(**a**–**f**) A variety of computed tomographic changes of maxilloturbinate region are labeled for pathologies related to acute/chronic infectious respiratory disease signs in Cats 6, 10 and 21. Window, level and laterality are also labeled for each section.

**Figure 10:**
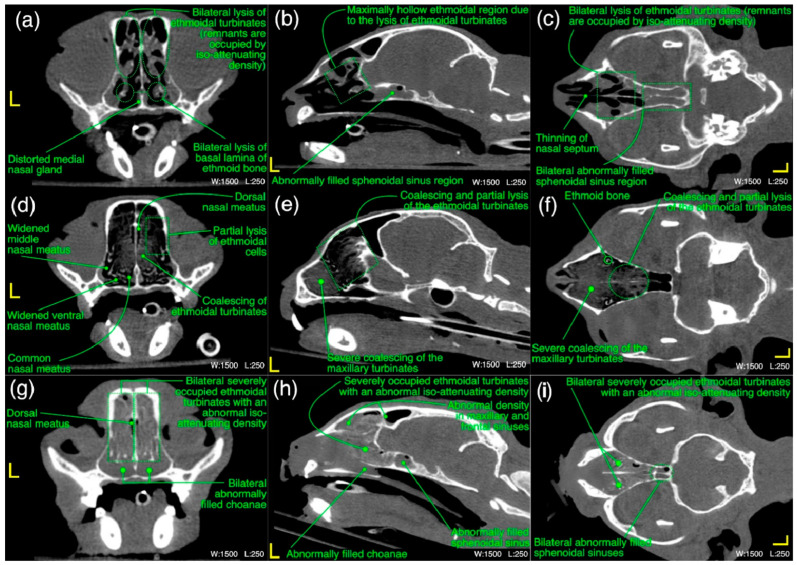
(**a**–**i**) A variety of computed tomographic changes of ethmoidal region are labeled for pathologies related to acute/chronic infectious respiratory disease signs in Cats 1, 6 and 18. Window, level and laterality are also labeled for each section.

**Figure 11:**
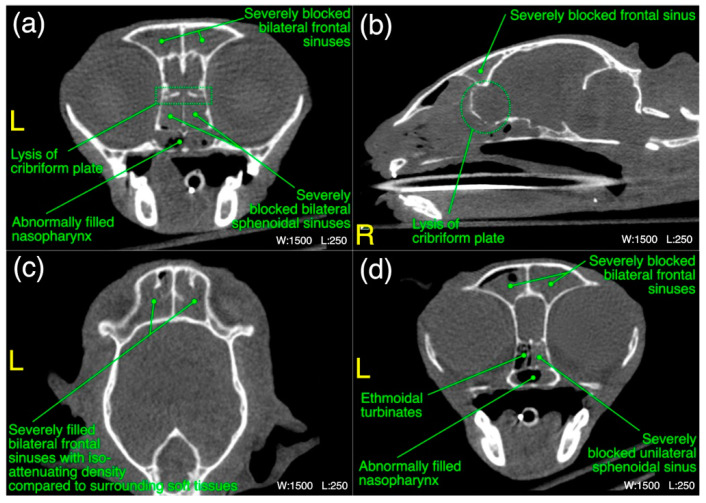
(**a**–**d**) A variety of computed tomographic changes of frontal sinus region are labeled for pathologies related to acute/chronic infectious respiratory disease signs in Cats 12 and 21. Window, level and laterality are also labeled for each section.

**Figure 12:**
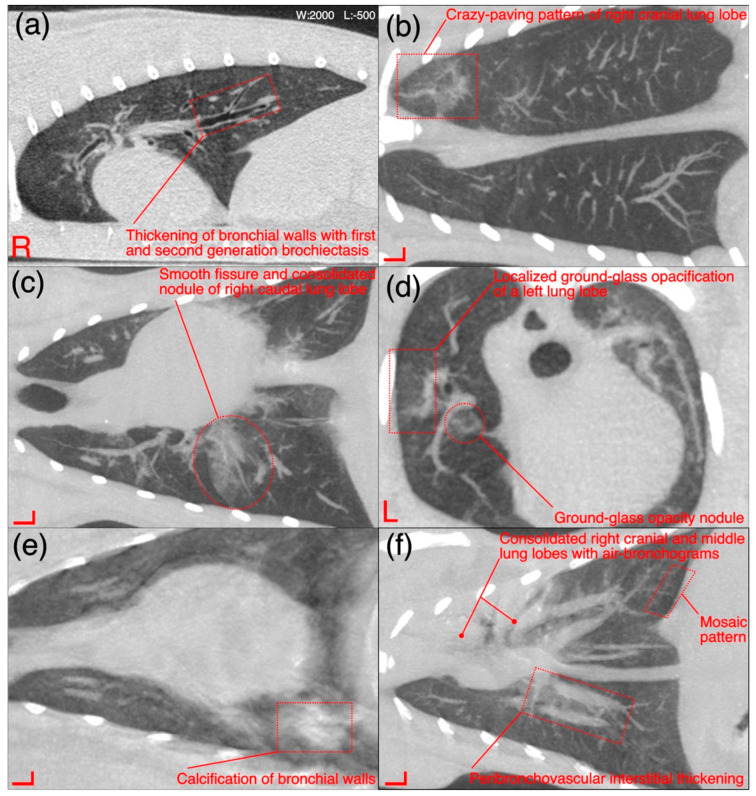
(**a**–**f**) A variety of computed tomographic changes of pulmonary parenchyma and its related vasculature are labeled for pathologies related to acute/chronic infectious respiratory disease signs in Cats 5, 8, 12 and 21. Window and level are same for all sections and laterality is also labeled for each side.

**Figure 13:**
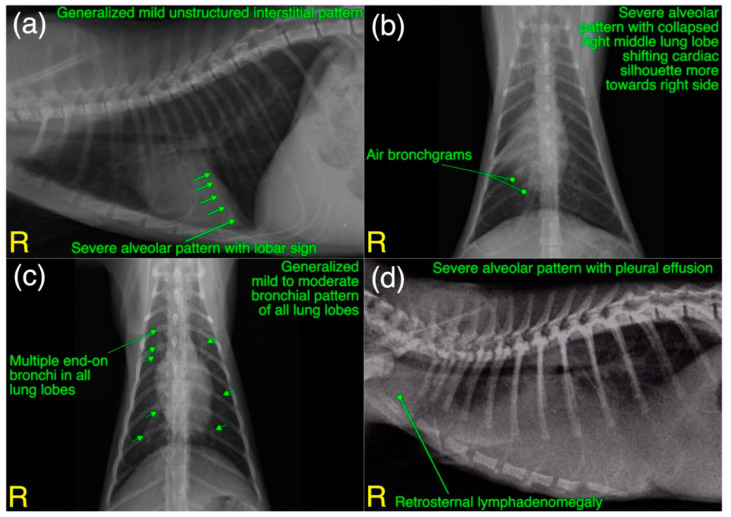
(**a**–**d**) A variety of radiographic changes (patterns) of thoracic cavity are labeled for pathologies related to acute/chronic infectious respiratory disease signs in Cats 15, 18 and 25. Laterality is also labeled for each radiograph.

**Figure 14:**
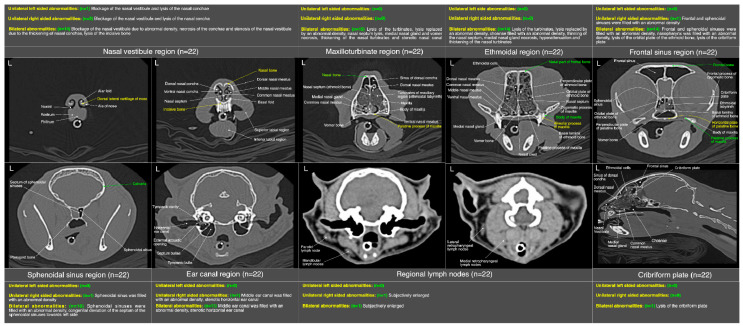
An illustrative summary of the localized computed tomographic abnormalities at focused regions of the skull in terms of their laterality in a total of 22 cats presented with acute/chronic infectious respiratory disease signs.

**Figure 15:**
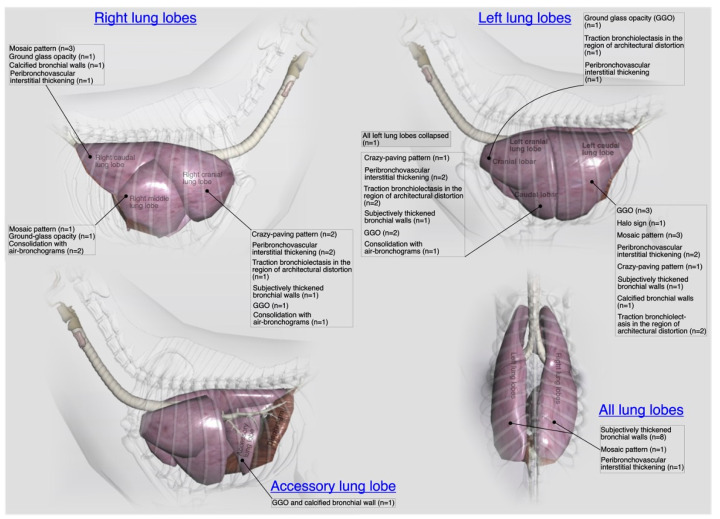
An illustrative summary of the localized computed tomographic abnormalities of the pulmonary parenchyma (individual lung lobes and lobules) and their relevant blood vessels in terms of their laterality in a total of 22 cats presented with acute/chronic infectious respiratory disease signs.

**Figure 16:**
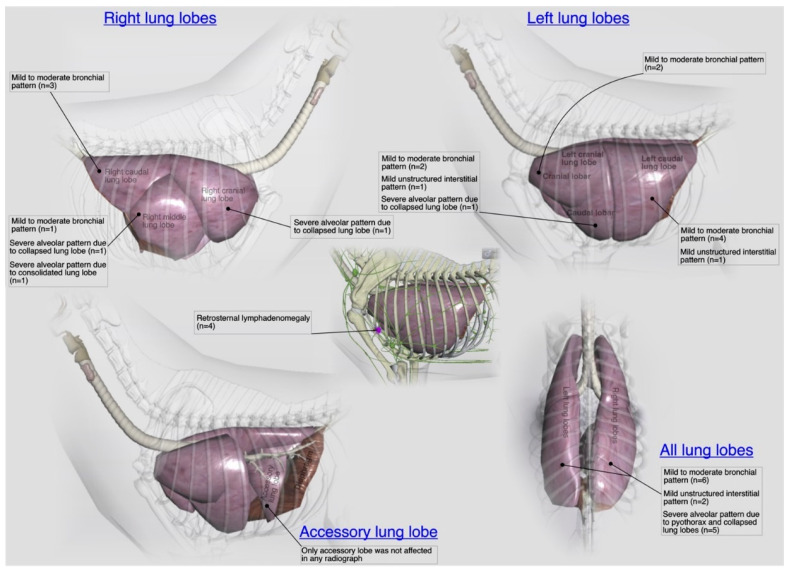
An illustrative summary of the localized radiographic abnormalities (patterns) of the pulmonary parenchyma (individual lung lobes and lobules) and their relevant blood vessels in terms of their laterality in a total of 27 cats presented with acute/chronic infectious respiratory disease signs.

The detailed radiological findings for individual case (22 cats’ CT scans and 27 cats’ thoracic radiographs) have been shown in Tables S1 and S2 (Supplementary Material). A regional summary of the various radiological abnormalities has been outlined in Table 9. A wide variety of computed tomographic and radiographic changes/patterns related to acute/chronic infectious respiratory disease signs of cats in present study have been demonstrated in detail from skull and thoracic radiological acquisitions (Figure 8, Figure 9, Figure 10, Figure 11, Figure 12 and Figure 13). Furthermore, illustrative summaries of the “localized radiological changes/patterns” of the focused skull and thoracic respiratory regions have been outlined in Figure 14, Figure 15 and Figure 16 with detailed anatomical annotations of the respiratory system. All CT scans were studied at 0.29–0.35 mm slab thickness for URT and 2–3 mm slab thickness for LRT examination.

The most prominent abnormality observed in nasal vestibule region was unilateral/bilateral mild to moderate pathological necrosis of the nasal conchae, noticed in 23% (*n* = 5) of cases, as shown in Figure 8d–f. Unilateral severe blockage of the nasal vestibule by an iso-attenuating density and bilateral severe blockage by an iso-/mixed-attenuating density [Figure 8g–i] was noticed in 18% (*n* = 4) cases for each category. Other most common abnormalities were bilateral moderate to severe thickening of the nasal conchae [Figure 8b], unilateral/bilateral stenotic nares [Figure 8a] and bilateral mild to moderate increase in the width of palatine fissure [Figure 8h] seen in 9% (*n* = 2), 14% (*n* = 3) and 9% (*n* = 2) cases, respectively. In terms of laterality and any affected structure(s), bilateral nasal vestibule abnormalities (Figure 14) were cumulatively recorded in 64% (*n* = 14) cases in localized CT scan abnormalities of this region.

In maxilloturbinate region, bilateral severely occupied nasal passage with an abnormal density excluding maxillary sinuses [Figure 9e,f] in 41% (*n* = 9) cases and distortion of the medial nasal gland [Figure 9c] in 36% (*n* = 8) cases followed by severe bilateral lysis of the maxillary turbinates [Figure 9c–f] in 32% (*n* = 7) cases and bilateral severely filled maxillary sinuses with an abnormal density [Figure 9c] in 27% (*n* = 6) cases were predominant findings. Mild lysis of the nasal septum was noticed in 27% (*n* = 6) cases. Distortion of the vomer bone [Figure 9d] of this region and unilateral/bilateral moderate lysis of the turbinates were noticed in 23% (*n* = 5) cases for each category. Moderate to severe thickening of the maxillary turbinates with/without hyper-attenuation and unilateral/bilateral severely stenotic nasal passage(s) [Figure 9a] due to severe thickening of the maxillary turbinates were noticed in 9% (*n* = 2) and 14% (*n* = 3) cases, respectively. In terms of localized findings of this region, again, bilateral abnormalities (Figure 14) were recorded in 68% (*n* = 15) cases, and unilateral left or right sides were not affected in any case.

In ethmoidal region, a bilateral moderate to severe lysis of the turbinates including basal laminae of the ethmoid bone [Figure 10a–c] in 55% (*n* = 12) cases was a predominant finding. Severe infiltration of the bilateral nasal passages and choanae of this region with an abnormal space occupying soft tissue/fluid density [Figure 10g–i] were seen in 32% (*n* = 7) of the cases. Distorted medial nasal gland [Figure 10a], bilateral moderate thickening of the ethmoidal turbinates with/without hyper-attenuation and partial lysis of the nasal septum [Figure 10c] were recorded in 27% (*n* = 6), 18% (*n* = 4) and 9% (*n* = 2) cases, respectively. As concerned with the localized findings of this region, here also bilateral abnormalities (Figure 14) were predominant findings and recorded in 64% (*n* = 14) cases and unilateral left or right side was not affected in any case.

In frontal sinus region, unilateral/mild to moderate bilateral infiltration of the frontal and sphenoidal sinuses with an abnormal soft tissue/fluid density was noticed in 45% (*n* = 10) and 32% (*n* = 7) cases, respectively. While severe infiltration of these sinuses with an abnormal soft tissue/fluid density [Figure 11a–d] was recorded in 9% (*n* = 2) for frontal sinuses and 23% (*n* = 5) cases for sphenoidal sinuses. Lysis of the cribriform plate [Figure 11a,b] and surrounding bony structures was noticed in 5% (*n* = 1) cases from each category. A mild to moderate infiltration of the nasopharynx [Figure 11a,d] with an abnormal soft tissue/fluid attenuating density was noted in 9% (*n* = 2) cases. Emphysematous fascial planes of the neck region were also recorded in 9% (*n* = 2) cases. In the summary of localized CT scan abnormalities (Figure 14) of this region, bilateral frontal and sphenoidal sinuses were affected in 64% (*n* = 14) and 45% (*n* = 10) cases, respectively. Unilateral right side was affected in one case only for both sinuses of this region. On the other hand, left side was not affected in any case for both frontal and sphenoidal sinuses. A single case of bilateral cribriform lysis was also recorded for this region (Figure 14).

Thickening of the first/second/third generation bronchial wall(s) [Figure 12a] was the predominant finding in thoracic region and observed in 36% (*n* = 08) cases. Calcification of the bronchial walls [Figure 12e] was present in one case only. Mild mosaic pattern [Figure 12f] of any lung lobe(s) and mild to moderate peribronchovascular interstitium thickening [Figure 12f] was noticed in 18% (*n* = 4) cases for each category. A ground-glass opacification (GGO) pattern [Figure 12d] of any lung lobe was recorded in 23% (*n* = 5) cases. Mild crazy-paving pattern [Figure 12b], mild traction bronchiolectasis in any lung lobe, and consolidation of any lung lobe was noticed in 9% (*n* = 2) cases for each category. Other uncommon findings were collapsed lung lobe(s) and pneumomediastinum, recorded in a single case for each category. In the summary of localized CT scan findings of pulmonary parenchyma (Figure 15), subjectively thickened bronchial walls of all lung lobes 36% (*n* = 8) followed by GGO 14% (*n* = 3) and mosaic patterns of left caudal lung lobe 14% (*n* = 3) and mosaic pattern of right caudal lung lobe 14% (*n* = 3) were predominant findings.

In additional CT scan findings, severe infiltration of the bilateral middle ear canal with an abnormal density was noticed in 36% (*n* = 08) cases, while moderate bilateral infiltration was recorded in 14% (*n* = 3) cases. A subjective mandibular lymphadenomegaly was noticed in 9% (*n* = 2) of the cases. In the summary of localized CT scan abnormalities (Figure 14) of ear canal and regional lymph nodes, bilateral middle ear canal was affected in 50% (*n* = 11) cases, while bilateral mandibular lymphadenomegaly was recorded in one case only. On the other hand, Unilateral right-sided abnormality of the middle ear canal and mandibular lymph node was recorded in one case only. Abnormality of unilateral left-sided ear canal and regional lymph node was not recorded in any case.

A total of 27 (22 from shelters and 5 privately owned) cats went through radiographic exams to investigate clinical signs related to acute/chronic infectious respiratory disease(s). Radiographically, liver silhouette was noticed beyond the costochondral junction with well tapered edges in 56% (*n* = 15) cases. A mild to moderate bronchial pattern [Figure 13c] and severe alveolar pattern [Figure 13a,b,d] of any lung lobe were noticed in 48% (*n* = 13) and 26% (*n* = 7) cases, respectively. A disturbance in caudal vena cava to descending aorta ratio (normal=0.77±0.2 for DSH cats) was calculated in 22% (*n* = 6) cases. Moderate to severe pleural effusion (19% (*n* = 5)), as shown in Figure 13d and retrosternal lymphadenomegaly (15% (*n* = 4)) were observed only in privately owned cats. A generalized mild to moderate interstitial pattern [Figure 13a] was also noticed in 14% (*n* = 3) cases. In localized radiographic findings, as shown in Figure 16, pathological changes affected all lung lobes more commonly compared to individual lung lobe and/or lobar from any side. In shelters’ cats mild to moderate bronchial pattern of all lung lobes was recorded in 22% (*n* = 6) cases followed by left 15% (*n* = 4) and right 11% (*n* = 3) caudal lung lobes with similar abnormal pattern. Only accessory lung lobe abnormality was not noticed in any radiograph of cats presented with acute/chronic infectious respiratory disease signs.

### 3.7. Pathological Findings

A total of 23 (22 from shelters and 1 privately owned) cats presented with an acute/chronic infectious respiratory disease signs underwent a detailed post-mortem examination to characterize the gross lesions and subsequent histopathological findings, particularly with reference to the focused bacteria. Grossly, lung tissue showed variable sized areas of consolidation in different lobes centered on bronchi in four animals. Congestion was noticed in 19 animals which varied from mild to moderate in seven cases and moderate to severe in 12 cats. One of the cats (Cat 25) tested positive for pulmonary form of the *R. equi*, while *K. pneumoniae* was isolated from trachea of the Cat 1 and 2 presented with acute/chronic infectious respiratory disease signs.

**Figure 17:**
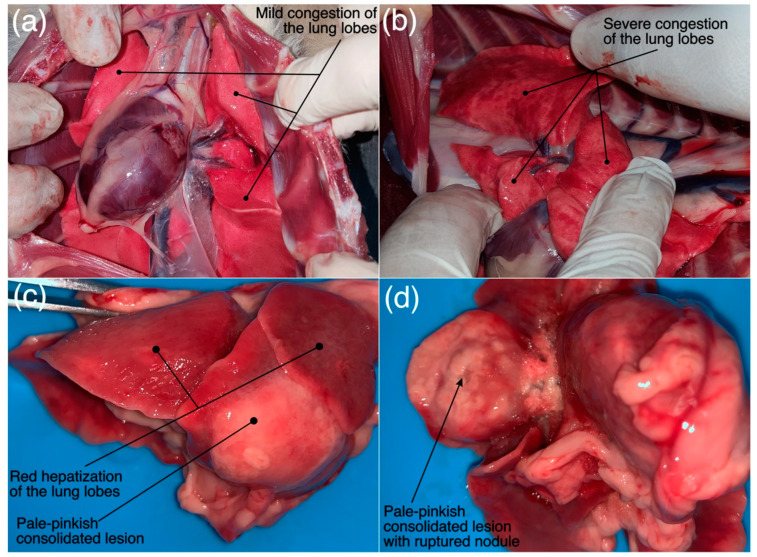
(**a**–**d**) Gross pathological findings (labeled) of (**a**) Cat 1 positive for FCoV and FCV antibodies and (**b**) Cat 2 positive for FIV, FCoV, FCV and FHV antibodies, presented with acute/chronic infectious respiratory disease signs and (**c**,**d**) Cat 25 diagnosed with pulmonary form of the feline rhodococcosis.

The thoracic cavity of the *R. equi* positive cat was filled with purulent material and right lung showed adhesions with the thoracic wall. The lungs were hepatized and had dark red discoloration and were firm in consistency, as shown in Figure 17c,d. Fibrinopurulent exudate was adhered to the lungs’ surfaces. Cut surface of the lungs demonstrated exudation of the purulent material from the bronchi and bronchioles. Middle lobe of right lung showed a raised pale-yellow solitary nodular lesion and was consolidated as well, as shown in Figure 17d. A single 1.5 × 1 × 0.4 cm enlarged retrosternal lymph node was also noticed during thorax exploration. Liver had mild to moderate congestion and intestines did not reveal any gross abnormality. On the other hand, Cat 1 and Cat 2 had congested lungs [Figure 17a,b] and liver without any obvious gross abnormality of the kidney, spleen and intestines.

A total of 65% (*n* = 15) cats had moderate to severe congestion including oedema of the lung tissues with/without bronchopneumonia findings (heavy infiltration of the inflammatory cells in alveolar spaces and bronchial spaces with necrotic and degenerated changes in epithelial cells of the lung tissues). Severe bronchopneumonia was noticed in 17% (*n* = 4) cats presented with acute/chronic infectious respiratory disease signs and a concurrent severe congestion with edematous fluid was noticed in two cases from this group. Moderate bronchopneumonia findings were recorded in 13% (*n* = 3) cases and a concurrent severe congestion with edematous fluid was noticed in two cases from this group also, while mild bronchopneumonia findings were noted in 9% (*n* = 2) cases.

**Figure 18:**
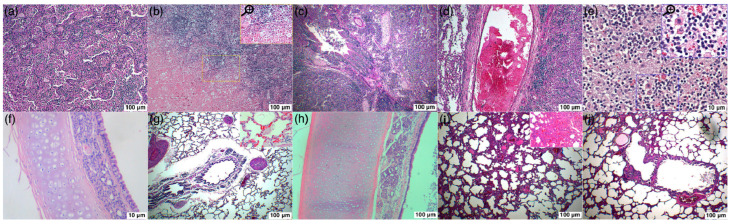
(**a**–**j**) Bronchopneumonia due to *R. equi* in Cat 25 (**a**–**e**). Cat 01 (**f,g**) positive for FCoV and FCV antibodies and Cat 02 (**h**–**j**) positive for FIV, FCoV, FCV and FHV antibodies were presented with acute/chronic infectious respiratory disease signs. *K. pneumoniae* was isolated from trachea of both cats. Photomicrograph (**a**) indicates severe infiltration of the mononuclear and polymorph inflammatory cells in alveolar spaces; (**b**) coagulative necrosis in lung tissue; (**c**) severe infiltration of the alveolar spaces with mononuclear inflammatory cells; bronchial lumen was infiltrated with inflammatory exudate, proteinaceous material and sloughed epithelial cells; necrosis and degeneration of the bronchial epithelium and severe infiltration of the peri-bronchial spaces with mononuclear inflammatory cells; (**d**) severely engorged arterial blood vessel with blood and necrosis and severe infiltration of the inflammatory cells in the surroundings; (**e**) severe infiltration of the alveolar spaces with mononuclear inflammatory cells especially macrophages and lymphocytes highlighted in magnified area; (**f**) normal trachea (**g**) relatively normal bronchus and alveolar spaces with congested blood vessels in surrounding and the highlighted area at the upper right corner shows acute congestion (mild) of the lung tissue being examined at higher magnification; (**h**) normal trachea with mildly sloughed epithelial cells in tracheal lumen; (**i**) severely congested and edematous alveolar walls and interstitial spaces; (**j**) relatively normal section of the lung tissue with mild congestion and edema of the alveolar walls and interstitial spaces. (H&E: Hematoxylin and Eosin; 40×–400×).

**Figure 19:**
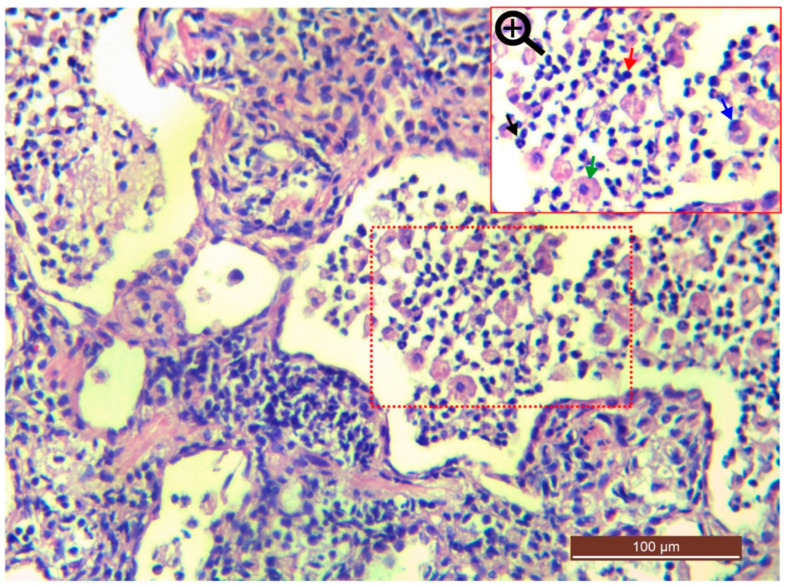
Bronchopneumonia due to *R. equi*. Photomicrograph of Cat 25 indicates exudate (combination of inflammatory cells and proteinaceous material) in the alveoli. The focused section (red dotted square) has been magnified and indicates a variety of cellularity such as macrophage (green arrow), neutrophil (black arrow), lymphocyte (red arrow) and plasma cell (blue arrow).

In focused bacteria cases, histopathological sections of the Cat 25 [Figure 18a–e] revealed the presence of exudate in the bronchi, bronchioles and alveoli. The exudative was comprised of a mixed population of inflammatory cells i.e., neutrophils, lymphocytes, plasma cells and macrophages, along with proteinaceous material, as shown in Figure 19. The overall examination showed that macrophages and lymphocytes were dominating cell types. The respiratory epithelium of the bronchi and bronchioles showed focal sloughing/degeneration. An area of coagulative necrosis was noticed in the lung tissue [Figure 18b]. Proliferation of type 2 pneumocytes were noticed only in a small area and most of the tissue reaction was centered on the bronchioalveolar section. On the other hand, histopathological sections of the Cat 1 and Cat 2 revealed mild and severe congestion respectively as the predominant finding with normal trachea, as shown in Figure 18f–j.

## 4. Discussion

There are many bacterial, viral, fungal, and protozoal causes of feline respiratory infections. In present study, all cats were recruited based on the criteria of acute/chronic infectious respiratory disease signs. Signalment and historical parameters such as age, sex, breed, neutering and vaccination status, lifestyle and positive pathogen status (if involved) have been studied extensively in previous cat studies to evaluate their relevance with the infectious respiratory tract disease [18,19,20].

Several studies have reported kittens and younger cats at greater risk of developing respiratory tract infections specifically due to viral and bacterial agents including *R. equi* [3,20]. However, in a previous study higher risk of URT infections have also been calculated for the cats with eleven years age group or older compared to the young adults [21]. In present study, all cats were DSH and manifestation of the infectious respiratory disease signs in relation to the known age group were in agreement with the previous cat studies where younger age group reported at higher risk for bacterial (including focused bacteria) and/or viral etiologies. Furthermore, a higher incidence of infectious respiratory disease due to FHV-1 and FCV in DSH cats has been reported in a previous cat study [22]. In cases of respiratory tract infection due to viral agents, FCV affects predominantly juveniles and young adults without any predilection to breed and sex. Similarly, FHV-1 can affect any age, breed and sex, but kittens up to the age of six months are over-represented for the respiratory infections due to this virus. However, most of the cases reported up to the age of 5 years in young adult cats with the signs of respiratory tract infections are due to viral organisms [23,24].

Most of the studies disagree about the reliability of sex predisposition as risk factor in URT infections [20,25]. On the other hand, in a comprehensive clinical review of LRT infections in cats, breed predilection was reported as none, but sex predilection was reported 2.4 times higher for males compared to the female population. This sex predilection was further exemplified by a retrospective study of fatal FHV-1 associated pneumonia, where all infected cats were male [2]. Sex predilection was not noticed in present study, probably affected by the selection of the cases with acute/chronic infectious respiratory disease signs.

Multi-cat household and/or free-roaming lifestyle have been reported as significant risk factor(s) to develop infectious respiratory disease, including pulmonary form of the feline rhodococcosis [3,18]. The risk calculated as high as 7.2 times for infectious URT disease in a multi-cat household [18]. In present study, this can be correlated to many factors, such as hygiene, exposure to etiological agents, mixing with co-habitant sick/carrier animals, vaccination status, and quality of life in relation to the exposed or confronted stress.

Clinical signs broadly overlap for all respiratory tract diseases of the cats and most of the time the disease is due to the involvement of multiple etiologic agents [1,26]. In most of the cases of URT infections specifically due to viral agents such as FCV and FHV-1, oral cavity is involved and manifests gingivitis, oral ulceration and faucitis as common clinical signs in URT disease [20,23]. Isolation or colonization of *R. equi* has never been reported from URT of the cats. However, its colonization as the resident aerobic microbiota of the healthy nasal cavity has been reported in a single human study [27]. In a previous retrospective study of nasal disease in cats due to various etiologies; nasal discharge (83%), URT noise (49%), sneezing (42%), ocular discharge (25%), dyspnea (18%), weight loss (16%), facial distortion (14%), lymphadenopathy (13%), lethargy (12%) and cough (06%) were recorded as prominent clinical signs [28]. In comparison to these clinical signs, the predominant findings of present study were lethargy (77%), low BCS (73%), and >5% dehydration (63%), which might be over-represented due to the predisposed and managemental conditions of the shelters, although the disease itself has a considerable part in these parameters. Nasal discharge was noticed in 100% cases. URT noise (63%), sneezing (55%), ocular discharge (50%), breathing abnormalities (37%), lymphadenopathy (5%) and coughing (9%) noticed in shelter cats, manifested acute/chronic infectious respiratory disease signs. Some of these clinical signs are regarded as pathogen incriminated clues, such as limping with dermatitis and oral ulcers are speculated due to FCV. On the other hand, oral ulceration with keratitis or corneal ulcer inclines clinicians to think about FHV-1. While conjunctivitis without nasal signs is usually correlated with *C. felis* and *Mycoplasma* spp. and coughing generally blamed due to *B. bronchiseptica* in cat specie [29].

Coughing, abnormal breathing patterns (dyspnea, tachypnea, orthopnea), abnormal respiratory sounds such as wheezing, crackling and inspiratory stridors, anorexia/hyporexia, sneezing, serous/mucoid oculonasal discharge, poor BCS/ weight loss, expiratory grunt, open-mouth breathing and pyrexia have been reported for a variety of LRT infections in cats due to bacterial, viral, fungal and protozoal causes including focused bacteria of this study [3,7,30]. In present study the percentage of the reported clinical signs specifically in pyothorax cases is very high and calculated as 100% for abnormal breathing patterns, abnormal respiratory sounds and anorexia/hyporexia, when compared with a recent retrospective study of cats with LRT disease due to *R. equi* [3]. In a previous retrospective study of 21 cats for LRT infections where pyothorax was not the focus, coughing was the most common (76%) clinical sign and presenting complaint as well, although dyspnea and/or tachypnea was also reported in a significant percentage (67%) of cases [7]. This pattern was not noticed in non-pyothorax LRT disease cases of this study, as coughing and breathing difficulties were noticed in 9% and 37% cases, respectively. Furthermore, lack of coughing and other relevant respiratory and systemic-illness signs does not rule out LRT disease from the differential diagnosis of a sick cat [2].

Since inflammation is the key process in infectious respiratory disease of cats, the hematological and biochemical parameters should suggest inflammatory changes such as leukocytosis, neutrophilia and changes in the acute phase proteins such as elevation of globulin [31,32]. A variety of non-consistent changes in erythrocyte and leukocyte parameters have been observed in previous cat studies of infectious respiratory disease. Not only the leukocytosis with neutrophilia or leukopenia with neutropenia, but normal leukocytes count has been recorded in fatal cases of the respiratory tract infections [3,7,33,34]. This study showed similar changes in hematology parameters. Since albumin and globulin are negative and positive acute phase proteins, respectively, changes in the globulin level are the most consistent findings especially in infectious LRT disease, observed in 87% samples of present study, which is causing low albumin to globulin ratio [2,3]. In cats, these changes have also been attributed to systemic diseases, hematological disorders, chronic infections, inflammatory process and neoplastic disorders [35]. The elevated urea in few cases can be correlated to prerenal azotemia due to dehydration and/or increased catabolism due to the fever or usage of the drugs such as tetracyclines [36].

In relation to the thrombocytopenia of 11 samples, it is important to highlight that the reference range for thrombocytes (300–700 × 10^9^ /L) has been generated by the hematology analyzer which is a default reference limit stored in the machine. The analyzed result of 10 cats can be still considered a normal figure for the cat specie, because in reference manual the normal limit for thrombocytes has been described as low as 150–400 × 10^9^ /L [37]. In eleventh cat, the thrombocytopenia of 84 × 10^9^/L can be related to any blood parasite or immune mediated disease, unfortunately not tested in this case.

Malaysia is a tropical country with a year-round hot and humid weather, that provides a suitable environment for the survival of ecto- and endoparasites. Surprisingly till date, feline lungworm has never been reported in Malaysian domestic or wild cats. Although, there are some emerging reports in relative species of felines and some other wildlife. In a recent conference proceeding, two cases of lungworms *(Metastrongylus* spp.) are reported in Asian palm civet and a wild boar [38,39]. Such occasional cases in different species doesn’t rule out the complete absence of such parasites from Malaysian environment. There is a dire need to conduct a survey study on a broader scale to investigate the presence of lungworm in Malaysian cat population.

The prevalence of viruses such as FeLV, FIV, FCoV, FCV and FHV is quite variable worldwide in shelter and client-owned cat’s studies. In a previous and only study of semi-roamer, outdoor, >6 months old, clients-owned 55 Malaysian cats, the antigenic prevalence of the FIV antibody status and FeLV antigen status was 23.6% and 12.7% respectively, using commercial immunochromatographic test kits [40]. Furthermore, in a previous conference proceeding results of the first and only molecular study of 24 cats with URT disease in Malaysia, FHV-1 was confirmed through PCR in 45.8% (11/24) oropharyngeal swabs of the client-owned cats. None of the sample was positive for FCV with reverse transcription polymerase chain reaction (RT-PCR). Majority of the cats (23/24) were unvaccinated in sampled population [41]. In Malaysia, a maximum prevalence of the FCoV has been reported 84% through RT-PCR of rectal swabs or fecal samples of 44 clinically healthy cats from two different catteries [42]. Compared with this reported viral background in Malaysia, FCV trended entirely opposite in present study and 96% samples produced a high antibody titer. In present study, although investigation was not carried out through RT-PCR but still a high antibody titer in a significant number of shelter and client-owned cats with poor to no vaccination records might suggest that there is a need to re-establish the background on prevalence of the disease due to FCV for the local setup. The FCoV antibody results of the present study (100%) cannot be compared with RT-PCR antigen test. However, generally, the trend remained same, and a very high population of the cats is still encountering this virus; thus, the organism still should be considered ubiquitous. The percentage of FeLV antigen and FIV antibody titer (detected by commercial immunochromatographic test kits) has been reduced dramatically in sampled population of this study and calculated in 4% and 15% samples, respectively. In present study, although the antibody titer has been calculated through semi-quantitative IgG immunoassay but the cumulative percentage of the medium to high antibody titer of FHV-1 (52%) is almost similar to the previously reported percentage of the oropharyngeal samples of FURD cases. FCV and FHV-1 have been reported as etiologic agents in upper and lower respiratory tract disease of the cats [2,43]. Approximately 90% of all URT disease in cats is due to FCV and FHV-1 only [1]. FeLV and FIV maybe involved in predisposition of the upper or lower respiratory tract infection due to the suppression of immune system of the cat [2,44].

In past, a significant number of bacteria have been isolated from upper and lower respiratory tract of the cats investigated for infectious respiratory disease [2,19,43,45]. In relation to the focused bacteria of this study, out of 12 pyothorax cases only one pleural effusion sample was positive for *R. equi* and out of 34 URT swab samples only 2 samples were positive for *K. pneumoniae*. The isolation of *R. equi* from pleural effusion of a 3-months old kitten having no history of contact with equine concentrated places and the histopathological findings of its lung tissues are further endorsing the observations of Aslam et al. [3] that kittens up to the age of 10 months are more prone to this bacterium compared to adults and senior cats. The immune suppression might be the predisposing factor, as this kitten was positive for FCoV and FCV antibodies. Unfortunately, albumin to globulin ratio was not available for this case. Given the normal histopathological findings of the two cats’ trachea (one of the sampling sites), *K. pneumoniae* can be considered as a colonized bacteria or resident microbiota of the URT. In presumed scenario of this colonization, isolation of the hypermucoviscous colony as a resident microbiota or colonized bacteria in one of these two cases is alarming, because hypermucoviscous is a property of the hypervirulent strains of *K. pneumoniae* [46]. To the best of author’s knowledge, this is the first study in Malaysia where *K. pneumoniae* has been reported presumably a colonized bacterium (because animals were not healthy) from URT of the cats and molecular identification of the *R. equi* has been done from a pleural effusion sample of a kitten.

Diagnosis of a respiratory disease, especially due to an infectious etiology requires a combination of techniques including, hematology, serum biochemistry, commercial bacterial/viral antigen or antibody tests, culture and susceptibility, cytology, radiology (preferably CT scan where available, although conventional radiography can be also very helpful), rhinoscopy and bronchoscopy (where indicated), and histopathology [1,2,47]. In terms of radiology, CT scan is a preferred modality to assess nasal passages, dental disease involvement, sinuses, surrounding bony structures and pulmonary parenchyma, but this requires general anesthesia. On the other hand, conventional radiography underestimates the extent of disease especially in URT [1,47]. Three orthogonal views of the thoracic radiographs are considered essential diagnostic modality in evaluation of the lower airway and pulmonary parenchymal disease. Radiographic evidence of infectious pneumonia can appear as a focal, multifocal, or diffuse alveolar, bronchial and interstitial pattern either alone or in combination(s), with/without any evidence of pneumothorax, consolidation and nodules in pulmonary parenchyma. Early in the disease process infiltrates reveal an interstitial pattern. Hence, lack of any obvious or major changes in thoracic radiographs doesn’t rule out LRT infection [7,48].

In present study, the viral investigation and bacterial growth from 62% samples of URT and 38% samples of LRT on culture media signify that several primary or secondary invaders were involved in respiratory disease of the cats. Since, focus of the research was more towards *R. equi* and *K. pneumoniae* hence, relevant samples were processed further for isolation and identification by biochemical and molecular technique. A variety of radiographic changes have been noticed in pulmonary parenchyma and thoracic cavity of the cats recruited in this study and their radiographic patterns are consistent with previous cat studies of LRT infections [7]. The presence of mild to moderate bronchial pattern in 48% radiographs (diffuse pattern more common in localized findings) and severe alveolar pattern in 26% radiographs is consistent with the histological diagnoses of bronchitis, bronchointerstitial pneumonia or bronchopneumonia, as shown in pathology results of this study. Such histological correlation of the radiographic findings has been tested in past also by Foster et al. [7] for LRT infections of cats. Furthermore, radiographic findings such as severe pleural effusion and enlargement of the retrosternal lymph nodes of the single pyothorax case due to *R. equi* are in complete agreement with the recent retrospective study of cats with pulmonary form of the feline rhodococcosis [3].

In a previous study, comparing the CT appearance of nasal disease due to neoplasia or inflammatory conditions in cats identified that many findings overlapped between these conditions. However, lysis of the nasal and surrounding bony structures, nasal turbinates and opacification of the sinuses were more pronounced with primary neoplasia of the nasal cavity [49]. Rostral nasal vestibule findings are not discussed in detail in two previous comprehensive cat studies, comparing neoplasia with inflammatory causes of the sinonasal disease, because epicenter of the disease “maxilloturbinate” remain common with all etiologies [15,49]. In present study, necrosis of the nasal conchae (23%) and unilateral (18%) or bilateral (18%) severe blockage of the nasal vestibule due to an abnormal iso-/mixed-attenuating density is the prominent feature of the disease due to any infectious etiology in rostral region. This is the first study where epicenter is investigated in relation to just infectious disease of the nasal cavity without any comparison with any neoplastic condition. Unlike previous two studies, maximum cases (55%) of bilateral moderate to severe lysis were noticed in ethmoidal turbinates followed by bilateral severe lysis of the maxilloturbinates in 32% cases. Although, there are another 23% cases where moderate unilateral or partial bilateral lysis has been recorded, hence, cumulative lysis of any degree was calculated as 55% in maxilloturbinate region. These findings might suggest that, in terms of infectious etiology of the sinonasal disease it may not be easier to refine differentials for a particular set of the diseases, as the pattern is quite variable from previous cat studies where multiple etiologies have been investigated and compared with neoplasia for CT changes. Furthermore, Infiltration of the meatuses at maxilloturbinate and ethmoidal region and effacement of the turbinates with an abnormal fluid/soft tissue density was noticed in 59% and 50% cases, respectively. Similar infiltrative findings have been reported by Tromblee et al. [49] in non-neoplastic nasal disease of the cats.

An abnormal infiltrative opacity (predominantly iso-attenuating) in maxillary, frontal and sphenoidal sinuses was recorded in an overall 41%, 54% and 55% cases, respectively. While severe degree of bilateral infiltration of sinus of dorsal conchae, frontal sinuses and sphenoidal sinuses were recorded in 27%, 9%, and 23% cases, respectively. In comparison with a previous study, the infiltration of the soft tissue/fluid attenuation was reported in 39% cases for frontal sinuses and 32% cases for sphenoidal sinuses in non-neoplastic nasal disease [49]. Contrary to this, Schoenborn et al. [15] reported much higher percentage of involvement of frontal and sphenoidal sinuses and calculated in 68% cases for both regions in non-neoplastic nasal disease. In present study, both regions are involved in almost 55% of cases. While previous studies didn’t report any prevalence of involvement for maxillary sinuses (sinus of dorsal conchae) [15,49]. A mild degree of lysis of nasal septum at maxilloturbinate region in 23% cases and ethmoidal region in 9% is correlated with previous reported changes in non-neoplastic nasal disease (rhinitis) group [15]. The prevalence of involvement of medial nasal gland (distorted) has not been reported in previous cat studies investigated nasal disease due to neoplastic or non-neoplastic (infections or inflammatory or mixed) etiologies [15,49]. Involvement of calvarium was not seen in any case of this study, similar observations were reported for infectious/inflammatory etiologies of nasal disease in previous cat studies [15]. While, evidence of unilateral/bilateral otitis media in significant number of cases and lysis of the paranasal bones, vomer and cribriform plate in minimal cases are quite parallel to the conclusion of previous cat studies when compared such changes in neoplastic and non-neoplastic nasal disease [15,49].

**Figure 20:**
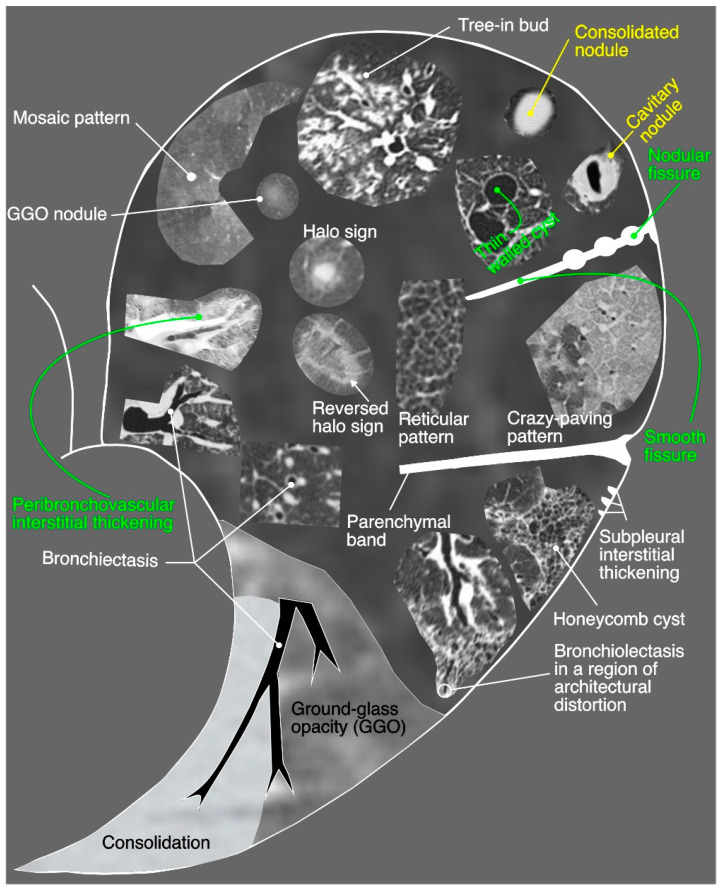
An illustrative sketch of the actual computed tomographic lung pathologies; can be seen in a variety of neoplastic and non-neoplastic pulmonary diseases of humans, dogs and cats.

A pattern-based approach to characterize the distribution and localization of the lesions is key to interpret lung CT studies. Four major patterns (increased attenuation, decreased attenuation, nodular patterns, linear patterns and mixed patterns) of a lung disease have been extrapolated from human studies and proposed in a recent guideline of thoracic CT scan for dogs and cats in the diagnosis of lung disease [12]. Same criteria were followed for interpretation of lung studies. These four major classes have further subclasses, and a variety of CT lung patterns (as shown in Figure 20) have been observed in cats recruited for present study.

Mild thickening of the bronchial walls, GGO and mild mosaic pattern of any lung lobe and peribronchovascular interstitial thickening are predominant findings in lung studies. Thickening of the first, second and third generation bronchial walls can be correlated with inflammatory changes of the bronchial tree, while perfusion abnormalities and patchy lung disease manifest mosaic pattern on CT scan. Peribronchovascular interstitial thickening belongs to the linear patterns’ class, and interstitial thickening is considered due to the infiltration of the cells, fibrous tissue, fluid, and increase in size and number of lymphatics or blood vessels around enveloping connective tissue of peribronchovascular regions [16]. The predominant bronchial pattern along with GGO and mosaic pattern indicate that bronchopneumonia is the more likely disease in recruited shelter cats compared with only bronchial or interstitial pneumoniae. The CT findings of the lungs are also consistent with radiographic and histological findings of this study. Although, LRT disease was not so severe in recruited shelter cats but the CT scan findings of the pulmonary parenchyma and thoracic cavity are closer to the previous human studies, reported infectious pneumonia findings due to a variety of bacterial and viral etiologies [50,51,52].

## 5. Conclusions

Infectious respiratory disease is a complex illness in cats, predominantly unvaccinated kittens and young adults, especially those kept in multi-cat household or shelter environment because of the involvement of multiple bacterial and viral organisms as primary or secondary invaders. Clinicians should not preclude feline rhodococcosis from differentials, especially in kittens with pyothorax and less than one year of age. A consistent manifestation of the pyothorax in *R. equi* cases can be due the poor to no response of the routine antibiotics used as empirical regimen for respiratory illness and chronicity of the infection. Unlike *R. equi*, *K. pneumoniae* has the potential to colonize in URT which might disseminate further to cause LRT disease in cats. Epidemiological studies needed for the Malaysian environment to establish the role of viral agents in pathophysiology of the disease. Advanced molecular techniques and radiological features are necessary to refine differentials in the management of infectious upper or lower respiratory tract disease. Pathogenesis of the disease is never reported for cat species, and virulence typing is not established yet for the strains isolated from pyothorax samples of Malaysian cats. Viral results of this prospective investigation revealed that prevalence of the infection should be re-established for the local setup of Malaysia.

## Figures and Tables

**Table 1:** The sequences, nucleotide positions and expected product size of primers designed to amplify specific partial sequences of targeted genes of *K. pneumoniae subsp. pneumoniae* and *R. equi*.

Oligonucleotide	Sequence	Nucleotide Position	Expected Product Size	Reference
*Klebsiella pneumoniae* (16S rDNA gene)				
KP16F	GCAAGTCGAGCGGTAGCACAG	50-70	260 bp	[13]
KP16R	CAGTGTGGCTGGTCATCCTCTC	279-309		
***Rhodococcus equi* (choE gene)**				
COX-F	GTCAACAACATCGACCAGGCG	1221-1241	959 bp	[14]
COX-R	CGAGCCGTCCACGACGTACAG	2160-2180		

**Table 2:** PCR reactions and gel electrophoresis conditions for the detection and/or confirmation of the *K. pneumoniae* and *R. equi* from cats.

Cycling Conditions	Reaction Mixture	Analysis
*Klebsiella pneumoniae*		
95 °C for 3 min [95 °C for 45 s, 58 °C for 45 s, 72 °C for 1 min] × 28 72 °C for 5 min	25 µL	1.5% gel 100 V for 30 min
***Rhodococcus equi***		
95 °C for 5 min [95 °C for 1 min, 57 °C for 1 min, 72 °C for 1 min] × 30 72 °C for 10 min	25 µL	1.5% gel 80 V for 90 min

**Table 3:** Clinical findings in shelter (*n* = 22) and privately owned cats (*n* = 12) manifesting acute/chronic infectious respiratory disease signs.

Parameters for Shelters’ Cats (*n* = 22)	*n* (%)
Lethargy	17 (77)
Low body condition score (BCS) (≤2.5)	16 (73)
Dehydration (>5%)	14 (63)
Stertorous respiratory sound	14 (63)
Sneezing	12 (55)
Serous nasal discharge	12 (55)
Unilateral or bilateral epiphora	11 (50)
Mucoid or mucopurulent nasal discharge	10 (45)
Oral ulceration	08 (36)
Encrustation of eyelids and/or external nares	08 (36)
Tachypnea	08 (36)
Fever	07 (32)
Conjunctivitis	06 (27)
Orthopnea	05 (23)
Dyspnea	03 (14)
Coughing	02 (09)
Mandibular lymphadenomegaly	01 (05)
**Parameters for privately owned pyothorax cats (*n* =** **12)**	
Respiratory distress	12 (100)
Abnormal respiratory sound such as crackles/absent lung sound	12 (100)
Anorexia/Hyporexia	12 (100)

**Table 4:** Hematological findings for a total of 22 (19 shelter and 3 privately owned) cats manifested acute/chronic infectious respiratory disease signs.

Sample ID	RBC (×10^12^/L)	Hb (g/L)	PCV (L/L)	MCV (fL)	MCHC (g/L)	WBC (×10^9^/L)	Band N (×10^9^/L)	Neut (×10^9^/L)	Lymp (×10^9^/L)	Mono (×10^9^/L)	Eosin (×10^9^/L)	Baso (×10^9^/L)	PLT (×10^9^/L)	P. Protein (g/L)
**Ref. Range**	05–10	80–150	0.24–0.45	39–55	300–360	5.5–19.5	<0.30	2.5–12.5	1.5–7.0	0.20–0.80	0.1–1.5	Rare	300–700	60–80
**Cat 01**	6.73	93	0.30	45	310	**18.37**	**0.55**	**15.61**	**0.37**	**1.84**	0.00	0.00	**123**	**98**
**Cat 02**	**11.12**	133	**0.44**	**40**	**302**	9.12	0.27	7.02	**1.09**	**0.73**	0.00	0.00	**264**	**112**
**Cat 03**	**4.80**	**82**	**0.26**	**54**	**661**	8.33	0.08	**3.00**	4.25	0.42	0.58	0.00	335	70
**Cat 04**	6.72	111	0.34	51	456	10.48	0.10	7.44	2.31	0.52	0.10	0.00	217	86
**Cat 05**	**4.94**	**73**	**0.23**	47	317	16.96	**0.34**	10.68	4.41	0.51	1.02	0.00	**248**	**92**
**Cat 06**	7.06	104	0.32	45	325	**21.06**	**0.42**	**15.37**	2.53	0.63	**2.11**	0.00	408	72
**Cat 07**	**5.15**	96	0.30	**58**	320	15.61	0.16	10.77	3.43	0.47	0.78	0.00	334	**94**
**Cat 08**	**5.98**	94	0.31	52	**303**	**21.48**	**0.43**	**14.61**	3.01	**0.86**	**2.58**	0.00	401	**78**
**Cat 09**	6.29	103	0.31	49	332	**20.14**	**0.40**	**13.49**	4.03	0.60	**1.61**	0.00	388	**64**
**Cat 10**	**5.31**	**87**	0.28	**53**	311	**29.32**	**0.88**	**18.18**	4.69	**1.47**	**4.10**	0.00	**269**	74
**Cat 11**	**5.86**	104	0.31	**53**	335	**23.22**	**0.46**	**16.95**	**1.86**	**1.16**	**2.79**	0.00	**225**	70
**Cat 12**	**5.82**	91	0.28	48	325	**22.50**	**0.45**	**14.40**	4.50	**1.35**	**1.80**	0.00	**84**	70
**Cat 13**	**5.63**	**85**	0.27	48	315	**0.52**	TFC	TFC	TFC	TFC	TFC	TFC	**160**	68
**Cat 14**	**5.92**	**86**	0.27	46	319	**5.95**	0.06	**2.14**	3.33	**0.18**	0.24	0.00	320	**62**
**Cat 15**	**4.09**	**67**	**0.21**	51	319	**26.06**	**0.52**	**12.51**	3.13	0.52	**9.38**	0.00	**245**	72
**Cat 19**	**5.46**	**87**	**0.26**	48	335	12.78	0.13	7.03	3.83	**0.26**	**1.53**	0.00	506	68
**Cat 20**	7.76	111	0.33	43	336	14.02	0.14	10.66	**1.68**	**1.12**	0.42	0.00	**190**	**100**
**Cat 21**	8.45	116	0.35	41	331	12.32	0.12	7.39	3.94	**0.74**	0.12	0.00	**203**	**86**
**Cat 22**	7.58	123	0.38	50	324	**21.66**	**0.43**	**15.60**	3.90	**1.52**	0.22	0.00	390	**82**
**Cat 24**	**4.64**	**63**	**0.17**	**36.2**	**375**	9.39	**susp**	**0.85**	**7.69**	**0.71**	0.08	--	332	--
**Cat 25**	6.61	**78**	**0.23**	**34.9**	338	**22.21**	**susp**	6.22	**14.68**	0.60	0.63	0.08	472	--
**Cat 26**	7.16	100	0.28	**39**	**357**	10.70	0.11	7.38	2.35	0.32	0.54	--	391	**110**

Bold values in red color are above the reference range; bold values in blue color are below the reference range; bold values in bold green color are falling at the lowest reference limit; bold values in brown color are falling at upper most reference limit; values in black color are well within reference range; RBC = red blood cells; Hb = hemoglobin; PCV = packed cell volume; MCV = mean corpuscular volume; MCHC = mean corpuscular hemoglobin concentration; WBC = white blood cells; Band N = band neutrophils; Neut = segmented neutrophils; Lymp = lymphocytes; Mono = monocytes; Eosin = eosinophils; Baso = basophils; PLT = platelets; P. Protein = plasma proteins; TFC = too few cells.

**Table 5:** Serum biochemistry findings for a total of 23 (22 shelter and 01 privately owned) cats manifested acute/chronic infectious respiratory disease signs.

Sample ID	ALP (U/L)	ALT (U/L)	GGT (U/L)	TP (Serum) (g/L)	ALB (g/L)	GLOB (g/L)	A:G (Unit)	TBIL (µmol/L)	Urea (mmol/L)	Creat (µmol/L)	Na^+^ (mmol/L)	K^+^ (mmol/L)	Cl^−^ (mmol/L)
Ref. Range	<80	10–90	<6.0	55–75	25–40	25–45	0.5–1.4	1.7–17	3–10	60–193	146–156	3.9–5.5	110–132
Cat 01	7	26	<2	**82.4**	**25.7**	**56.7**	**0.5**	**16.4**	**35.2**	159	–	–	–
Cat 02	6	71	<2	**101.8**	31.9	**69.9**	**0.5**	**47.2**	**31.3**	118	–	–	–
Cat 03	13	36	4	68.6	**23.6**	**45.0**	**0.5**	2.3	**10.2**	126	–	–	–
Cat 04	14	64	<2	**98.0**	**24.6**	**73.4**	**0.3**	**1.5**	8.1	100	–	–	–
Cat 05	20	36	<2	**78.8**	29.4	**49.4**	0.6	**1.2**	**11.0**	104	–	–	–
Cat 06	15	38	<2	**96.2**	**22.4**	**73.8**	**0.3**	**1.1**	8.0	115	–	–	–
Cat 07	7	48	<2	**85.3**	**24.5**	**60.8**	**0.4**	**0.9**	7.6	116	–	–	–
Cat 08	19	63	<2	72.3	31.5	40.8	0.8	**1.3**	8.3	101	–	–	–
Cat 09	12	39	<2	**75.4**	**21.4**	**54.0**	**0.4**	**1.6**	6.8	88	–	–	–
Cat 10	7	26	<2	**82.4**	**25.7**	**56.7**	**0.5**	16.4	**35.2**	159	–	–	–
Cat 11	14	37	4	**75.1**	**22.0**	**53.1**	**0.4**	**1.2**	6.4	115	–	–	–
Cat 12	10	36	<2	**74.5**	**23.3**	**51.2**	**0.5**	**1.3**	6.6	90	–	–	–
Cat 13	24	27	<2	**73.7**	28.0	**45.7**	0.6	1.9	5.9	80	–	–	–
Cat 14	15	51	<2	69.4	**24.5**	**44.9**	**0.5**	**1.5**	9.3	107	–	–	–
Cat 15	14	**90**	<2	**74.6**	**22.6**	**52.0**	**0.4**	**1.3**	9.3	**72**	–	–	–
Cat 16	2	**107**	<2	**93.7**	**24.2**	**69.5**	**0.3**	4.2	**24.5**	**72**	–	–	–
Cat 17	19	51	3	**73.1**	**25.7**	**47.4**	**0.5**	**1.3**	7.7	87	–	–	–
Cat 18	9	47	<2	**73.6**	**21.5**	**52.1**	**0.4**	**0.8**	7.6	**60**	–	–	–
Cat 19	8	54	3	72.4	**20.6**	**51.8**	**0.4**	**1.2**	7.8	75	–	–	–
Cat 20	14	50	5	**104.7**	**24**	**80.7**	**0.3**	2.2	**11.8**	129	–	–	–
Cat 21	3	41	<2	**86.3**	28.5	**57.8**	**0.5**	2.1	7.1	**71**	–	–	–
Cat 22	4	82	<2	**83.7**	**25.7**	**58**	**0.4**	2.3	8	80	–	–	–
Cat 26	12	51	–	**96.8**	**23.1**	**73.7**	**0.3**	–	6.0	**60**	150	5.3	112

Bold values in red color are above the reference range; bold values in blue color are below the reference range; bold values in green color are falling at the lowest reference limit; bold values in brown color are falling at upper most reference limit; values in black color are well within reference range; Na^+^ = Sodium; K^+^ = Potassium; Cl^-^ = Chloride; Creat = Creatinine; TBIL = Total bilirubin; ALT = Alanine transaminase; ALKP = Alkaline phosphatase; GGT = Gamma-glutamyl transferase; TP = Total protein; ALB = Albumin; GLOB = Globulin; A:G = Albumin: Globulin ratio.

**Table 6:** Viral screening results through qualitative snap tests and semi-quantitative IgG immunoassays in a total of 27 (22 shelter and 05 privately owned) cats manifested acute/chronic infectious respiratory disease signs.

Patient ID	FIV Antibody	FeLV Antigen	FCoV Antibody	FCV Antibody	FHV Antibody
Cat 01	Negative	Negative	Positive	High titer (5), VN 1:128	Negative(0), VN ≤ 1:2
Cat 02	Positive	Negative	Positive	High titer (6), VN ≥ 256	Medium titer (3), VN 1:16
Cat 03	Negative	Negative	Positive	High titer (6), VN ≥ 256	Negative(0), VN ≤ 1:2
Cat 04	Positive	Negative	Positive	High titer (6), VN ≥ 256	Low titer (1), VN 1:4
Cat 05	Negative	Negative	Positive	High titer (6), VN ≥ 256	Medium titer (3), VN 1:16
Cat 06	Negative	Negative	Positive	High titer (6), VN ≥ 256	Low titer (1), VN 1:4
Cat 07	Negative	Negative	Positive	High titer (6), VN ≥ 256	Low titer (2), VN 1:8
Cat 08	Negative	Negative	Positive	High titer (6), VN ≥ 256	Medium titer (3), VN 1:16
Cat 09	Negative	Negative	Positive	High titer (6), VN ≥ 256	Low titer (1), VN 1:4
Cat 10	Negative	Negative	Positive	High titer (6), VN ≥ 256	Medium titer (3), VN 1:16
Cat 11	Negative	Negative	Positive	High titer (6), VN ≥ 256	High titer (5), VN 1:64
Cat 12	Negative	Negative	Positive	High titer (6), VN ≥ 256	High titer (5), VN 1:64
Cat 13	Negative	Negative	Positive	High titer (6), VN ≥ 256	Negative(0), VN ≤ 1:2
Cat 14	Negative	Negative	Positive	High titer (6), VN ≥ 256	Medium titer (3), VN 1:16
Cat 15	Positive	Negative	Positive	High titer (6), VN ≥ 256	High titer (5), VN 1:64
Cat 16	Positive	Negative	Positive	High titer (6), VN ≥ 256	High titer (6), VN ≥ 1:128
Cat 17	Negative	Negative	Positive	High titer (5), VN 1:128	Negative(0), VN ≤ 1:2
Cat 18	Negative	Negative	Positive	High titer (6), VN ≥ 256	Low titer (2), VN 1:8
Cat 19	Negative	Positive	Positive	High titer (6), VN ≥ 256	High titer (4), VN 1:32
Cat 20	Negative	Negative	Positive	High titer (6), VN ≥ 256	Medium titer (3), VN 1:16
Cat 21	Negative	Negative	Positive	High titer (5), VN 1:128	High titer (5), VN 1:64
Cat 22	Negative	Negative	Positive	High titer (6), VN ≥ 256	Low titer (2), VN 1:8
Cat 23	Negative	Negative	Positive	High titer (6), VN ≥ 256	High titer (5), VN 1:64
Cat 24	Negative	Negative	Positive	High titer (6), VN ≥ 256	High titer (6), VN ≥ 1:128
Cat 25	Negative	Negative	Positive	High titer (4), VN 1:64	Negative(0), VN ≤ 1:2
Cat 26	Negative	Negative	Positive	Negative(0), VN ≤ 1:4	Low titer (1), VN 1:4
Cat 27	Negative	Negative	Positive	High titer (5), VN 1:128	Negative(0), VN ≤ 1:2

Values inside the brackets (n) indicate scale of the antibody titer; values in red color indicate high antibody titer or positive result for a qualitative snap test; values in brown color indicate medium antibody titer; values in green color indicate low antibody titer.

**Table 7:** Summary of the bacterial culture, biochemical and molecular confirmation results from URT and LRT samples in a total of 34 (22 shelter and 12 privately owned) cats manifested acute/chronic infectious respiratory disease signs.

Patient ID	Growth on Blood Agar		Growth on MacConkey Agar		Biochemical Confirmation for *K. pneumoniae* and *R. equi*		PCR Confirmation for *K. pneumoniae* and *R. equi*	
	URT Swab	LRT Swab	URT Swab	LRT Swab	URT Sample	LRT Sample	URT Sample	LRT Sample
Cat 01	+	–	+	–	+ (KP)	–	+ (KP)	–
Cat 02	+	–	+	–	+ (KP)	–	+ (KP)	–
Cat 03	–	–	–	+	–	+ (K.sp)	–	–
Cat 04	–	–	–	–	–	–	–	–
Cat 05	–	–	–	–	–	–	–	–
Cat 06	–	–	–	–	–	–	–	–
Cat 07	+	+	–	+	–	–	–	–
Cat 08	+	–	–	–	–	–	–	–
Cat 09	–	–	–	–	–	–	–	–
Cat 10	–	–	–	–	–	–	–	–
Cat 11	+	–	+	–	–	–	–	–
Cat 12	+	–	–	–	–	–	–	–
Cat 13	–	–	–	–	–	–	–	–
Cat 14	+	–	–	–	–	–	–	–
Cat 15	+	+	+	–	–	–	–	–
Cat 16	–	–	–	–	–	–	–	–
Cat 17	–	–	–	–	–	–	–	–
Cat 18	+	+	+	+	–	–	–	–
Cat 19	+	–	–	–	–	–	–	–
Cat 20	+	–	–	–	–	–	–	–
Cat 21	+	+	–	–	–	–	–	–
Cat 22	–	–	–	–	–	–	–	–
Cat 23	+	–	–	–	–	–	–	–
Cat 24	+	+	+	–	–	–	–	–
Cat 25	–	+	–	–	–	+ (RE)	–	+ (RE)
Cat 26	+	+	–	–	–	–	–	–
Cat 27	+	+	–	–	–	–	–	–
Cat 28	+	+	–	–	–	–	–	–
Cat 29	+	–	–	–	–	–	–	–
Cat 30	–	+	–	+	–	–	–	–
Cat 31	–	+	–	–	–	–	–	–
Cat 32	+	+	–	–	–	–	–	–
Cat 33	+	–	–	–	–	–	–	–
Cat 34	+	+	+	–	–	–	–	–

(KP) = Klebsiella pneumoniae; (K.sp) = *Klebsiella* spp.; (RE) = *Rhodococcus* equi.

**Table 8:** Summary of conventional biochemical tests, conducted to confirm the colonies of *K. pneumoniae* in Cat 01 and 02 and undifferentiated *Klebsiella* spp. in Cat 03 and *R. equi* in Cat 25, manifested acute/chronic infectious respiratory disease signs.

	Triple Sugar Iron				SIM			Citrate	Urease	Oxidase
	Slant	Butt	Gas	H_2_S	Sulphide	Indole	Motility			
Cat 01 Trachea	Alkaline	Acid	+	–	–	–	–	+	+	–
Cat 02 Trachea	Acid	Acid	+	–	–	–	–	+	+	–
Cat 03 Lungs	Acid	Acid	+	–	–	–	–	+	+	–
	**Catalase**			**Urease**			**Glucose**		**Nitrate reduction**	
Cat 25 Lungs	Positive			Positive			Negative		Variable	

**Table 9:** Summary of the radiological findings in a total of 27 cats investigated for acute/chronic infectious respiratory disease signs.

**Parameters**	**N (%)**
**Nasal vestibule (*n* =** **22)**	
Unilateral moderate to severe thickening of the nasal conchae	01 (05)
Bilateral moderate to severe thickening of the nasal conchae	02 (09)
Severe blockage of the bilateral nasal vestibule with iso-/mixed-attenuating density	04 (18)
Unilateral mild to moderate blockage of the nasal vestibule with iso-attenuating density	03 (14)
Unilateral severe blockage of the nasal vestibule with iso-attenuating density	04 (18)
Mild lysis of the surrounding bony structures	01 (05)
Unilateral/bilateral mild to moderate pathological necrosis of the nasal conchae	05 (23)
Bilateral mild to moderate increase in width of palatine fissure	02 (09)
Unilateral/bilateral stenotic and mildly blocked nares (nasal opening)	03 (14)
**Maxilloturbinate region (*n* =** **22)**	
Moderate to severe thickening ± hyper-attenuation of maxillary turbinates	02 (09)
Bilateral severe lysis of the turbinates	07 (32)
Unilateral/bilateral moderate lysis of the turbinates	05 (23)
Bilateral partially/unilaterally (mild to moderate) occupied nasal passage with an abnormal density at maxilloturbinate region excluding sinus of dorsal conchae	04 (18)
Bilateral severely occupied nasal passage with an abnormal density at the level of maxilloturbinate region excluding sinus of dorsal conchae	09 (41)
Partial (mild) lysis of nasal septum	06 (27)
Unilaterally/bilateral partially (moderate to severe) filled sinus of dorsal conchae	03 (14)
Bilateral severely filled sinus of dorsal conchae	06 (27)
Distortion/mild to moderate necrosis of the medial nasal gland	08 (36)
Distortion/mild lysis of the vomer at any point along the nasal passage of this region	05 (23)
Unilateral/bilateral severely stenotic maxillary nasal passage due to the thickening of maxillary turbinates	03 (14)
**Ethmoidal region (*n* =** **22)**	
Bilateral moderate to severe lysis of the turbinates including basal laminae of ethmoid bone	12 (55)
Bilateral moderate thickening ± hyper-attenuation of the ethmoidal turbinates including basal laminae of ethmoid bone	04 (18)
Severe infiltration of abnormal space occupying density in bilateral nasal cavity of this region	07 (32)
Mild to moderate infiltration of abnormal space occupying density in bilateral nasal cavity of this region	04 (18)
Mild lysis of nasal septum	02 (09)
Distorted/ moderate to severe necrosis of medial nasal gland	06 (27)
Severe infiltration of the choanae with abnormal fluid/soft tissue density	07 (32)
Mild to moderate infiltration of the choanae with abnormal fluid/soft tissue density	03 (14)
**Frontal sinus region (*n* =** **22)**	
Severe infiltration of the bilateral frontal sinuses with abnormal soft tissue/fluid density	02 (09)
Unilateral/mild to moderate bilateral infiltration of the frontal sinuses with abnormal soft tissue/fluid density	10 (45)
Severe infiltration of the bilateral sphenoidal sinuses with abnormal soft tissue/fluid density	05 (23)
Unilateral/mild to moderate bilateral infiltration of the sphenoidal sinuses with abnormal soft tissue/fluid density	07 (32)
Mild lysis of the cribriform plate	01 (5)
Mild to moderate infiltration of the nasopharynx with abnormal soft tissue/fluid density	02 (09)
Mild to moderate lysis of the surrounding bony structures	01 (05)
**Neck region (*n* =** **22)**	
Subcutaneous emphysema in fascial planes of the neck region	02 (09)
**Thoracic region (*n* =** **22)**	
Mild thickening of the first/second/third generation bronchial walls	08 (36)
Calcification of bronchial wall in any lung lobe(s)	01 (05)
Mild mosaic pattern in any lung lobe	04 (18)
Mild crazy-paving pattern	02 (09)
Mild to moderate peribronchovascular interstitial thickening	04 (18)
Ground glass opacification pattern in any lung lobe	05 (23)
Halo sign in any lung lobe(s)	01 (05)
Mild traction bronchiolectasis in any lung lobe(s)	02 (09)
Consolidation of any lung lobe(s)	02 (09)
Any collapsed lung lobe(s)	01 (05)
Pneumomediastinum	01 (05)
Megaesophagus	01 (05)
Congenital pectus excavatum	01 (05)
Congenital loss of unilateral diaphragmatic outline resulting into diaphragmatic hernia	01 (05)
**Additional findings from CT scans of skull, neck and thorax (*n* =** **22)**	
Severe infiltration of the bilateral middle ear canal (tympanic bulla and tympanic cavity) with an abnormal soft tissue/fluid density	08 (36)
Severe infiltration of the unilateral middle ear canal (tympanic bulla and tympanic cavity) with an abnormal soft tissue/fluid density	01 (05)
Moderate infiltration of the bilateral middle ear canal (tympanic bulla and tympanic cavity) with an abnormal soft tissue/fluid density	03 (14)
Subjectively enlarged mandibular lymph node(s)	02 (09)
**Radiographic findings [(*n* =** **27) 22 shelter cats +5 privately owned cats]**	
Mild to moderate bronchial pattern in any lung lobe(s)	13 (48)
Generalized mild to moderate unstructured interstitial pattern in any lung lobe(s)	03 (14)
Severe alveolar pattern with air bronchograms in any lung lobe(s)	07 (26) **
Mild vascular pattern including disturbance in caudal vena cava to descending aorta ratio (normal = 0.77 ± 0.2 for DSH cats) ^#^	06 (22)
Moderate to severe pleural effusion	05 (19)
Cavitary lesion/pneumatocele in any lung lobe(s)	02 (07)
Congenital pectus excavatum	01 (04)
Congenital loss of unilateral diaphragmatic outline resulting into diaphragmatic hernia	01 (04)
Liver silhouette beyond costochondral junction with well tapered edges	15 (56)
Retrosternal lymph node enlargement	04 (15) *
Deviated cardiac silhouette due to congenital malformations	02 (07)
Slight to significant gas opacity in cervical and/or thoracic esophagus	04 (15)

* number (%) indicates cases only from privately owned pyothorax cats; ** number (%) included 5 privately owned pyothorax cats, # normal value adapted from Vosugh & Nazem [17].

## Data Availability

All the relevant data related to this research has been provided in this article and in attached supplementary tables. The data related to this research is not published anywhere else.

## Supplementary Materials

The following supporting information can be downloaded at: https://www.mdpi.com/article/10.3390/microorganisms11030737/s1, Table S1: Detailed CT scan findings of in 22 cats presented with acute/chronic infectious respiratory diseas signs; Table S2: Detailed radiographic findings of in 27 cats presented with acute/chronic infectious respiratory diseas signs.
