# Supplementary material for: Clinicopathological and Radiological Features of Cats Presented with Infectious Respiratory Disease Signs: A Focus on Rhodococcus equi and Klebsiella pneumoniae
Source: Microorganisms. 2023 Mar 14;11(3):737. doi: 10.3390/microorganisms11030737 (PMC10057062; doi:10.3390/microorganisms11030737)
Supplement: Supplementary file 1 [file microorganisms-11-00737-s001.zip › microorganisms-2226145-supplementary.pdf]

Table S1: Detailed CT scan findings of in 22 cats presented with acute/chronic infectious respiratory diseas signs

	Nasal Vestibule	Maxilloturbinate Region	Ethmoidal Region	Frontal Sinus Region	Neck Region	Thoracic Region	Additional Findings
Cat 01	In axial, dorsal and sagittal pre-contrast soft tissue MPR, rough and spikey outline of the nasal planum and nares noticed, possibly due to the sticky material on nasal opening. Severely blocked bilateral nasal vestibule with mixed-attenuating density.	In dorsal, sagittal and axial pre-contrast bone and soft tissue MPR, bilateral complete lysis (severe) of the turbinates noticed in this region. A soft tissue attenuation filling the entirety of the bilateral nasal cavity including sinus of dorsal concha and dorsal and ventral nasal meatuses. Partially lysed (mild) ventral nasal septum and loss of the medial nasal gland. Soft tissue around the cavity was thickened, rough and irregularly marginated	In dorsal, sagittal and axial pre-contrast bone and soft tissue MPR, a bilateral curled shelf of turbinates can be appreciated as hyper-attenuated labyrinth, but a bilateral severe soft tissue attenuation was filling almost complete entirety of this region with mild air trappings in dorsal nasal meatus, dorsal aspect of the left nasal cavity and medio-middle aspect of the right nasal cavity. Partial loss (mild to moderate) of the turbinates were noticed in caudal aspect of the nasal cavity. Septum and vomer were still intact. Bilateral choanae was soft tissue density filled	In dorsal, sagittal and axial pre-contrast soft tissue MPR, rostral to caudal both frontal sinuses had an abnormal slight soft tissue attenuating patch (mild), although right side was affected more rostrally compared to left but major portion of the caudal aspect of the sinuses were clear from any obvious abnormality. Cranial nasopharynx and bilateral sphenoidal sinuses were partially (mild) filled with soft tissue attenuating density	In dorsal, sagittal and axial pre-contrast soft tissue MPR, a distinct subcutaneous emphysema was noticed around bilateral fascial planes of the neck region	In dorsal, sagittal and axial pre-contrast soft tissue and lungs MIP and MPR, multifocal mild mosaic pattern in right middle and left and right caudal lung lobes was noticed, no other obvious abnormality was noticed in pulmonary parenchyma, thoracic vasculature and airways. Pleural and mediastinal spaces including mediastinal structures were free from any obvious abnormality	In axial, dorsal and sagittal pre-contrast bone and soft tissue MPR, distinct patches of the soft tissue attenuation without any obvious attachment to body tissue were noticed in bilateral horizontal ear canals, indicating possible ceruminous wax. Bilateral tympanic bullae, septum bullae, tympanic cavity and musculotubal canals were free from any obvious abnormality. A subjectively enlarged (not measurable in pre-contrast reconstruction) right mandibular lymph node was noticed
Cat 02	In axial, dorsal and sagittal pre-contrast soft tissue MPR, rough, irregular and spikey outline of the nasal planum and nares was noticed, possibly due to the sticky material on nasal opening. Severely blocked bilateral nasal vestibule with mixed	In dorsal, sagittal and axial pre-contrast bone and soft tissue MPR, a bilateral complete lysis (severe) of the turbinates was noticed in this region. A soft tissue attenuation filling the entirety of the bilateral nasal cavity including sinus of dorsal concha and dorsal and ventral nasal meatuses. Partially lysed (mild) ventral nasal septum and loss of the medial nasal gland. Soft	In dorsal, sagittal and axial pre-contrast bone and soft tissue MPR, bilateral basal lamina of the ethmoid bone and few turbinates in dorsal aspect of the cavity could be appreciated as hyper-attenuated labyrinth, but a bilateral soft tissue attenuation was filling almost complete entirety of this region with few multifocal air trapping regions. A moderate to severe lysis of the turbinates was noticed in	In dorsal, sagittal and axial pre-contrast soft tissue MPR, rostral to caudal both frontal sinuses had an abnormal slight soft tissue attenuating patch, although right side was affected more rostrally compared to left but major portion of the caudal aspect the sinuses were clear from any obvious abnormality. Cranial nasopharynx and bilateral sphenoidal	In dorsal, sagittal and axial pre-contrast soft tissue MPR, a distinct subcutaneous emphysema was noticed around bilateral fascial planes of the neck region.	In dorsal, sagittal and axial pre-contrast soft tissue and lungs MIP and MPR, multifocal mild mosaic pattern in left and right caudal lung lobes was noticed. A massive almost 12mm pneumomediastinum strip noticed from cranial to caudal mediastinum especially in dorsal view. Mainstem bronchi, multiple end-on bronchi, and bronchioles were subjectively thickened, no	In axial, dorsal and sagittal pre-contrast bone and soft tissue MPR, bilateral tympanic bullae and tympanic cavities including musculotubal canals and cochlea were filled with soft tissue attenuating density, tympanic membrane could not be appreciated. Horizontal ear canal was clear and septum bullae was still intact.

	attenuating density.	tissue around the nasal cavity was thickened, rough and irregularly marginated.	this region. Septum and vomer bone were still intact and bilateral choanae was filled soft tissue attenuating density.	sinuses were partially (mild) filled with soft tissue attenuating density. A distinct subcutaneous emphysema was noticed around bilateral fascial planes of this region.		other obvious abnormality was noticed in pulmonary parenchyma, thoracic vasculature and airways. Mediastinal structures were free from any obvious abnormality	
Cat 03	In axial, dorsal and sagittal pre-contrast bone and soft tissue MPR, no obvious abnormality was noticed in nasal planum, nares, nasal conchae and nasal vestibule.	In dorsal, sagittal and axial pre-contrast bone and soft tissue MPR, bilateral sinus of dorsal conchae, nasal turbinates, dorsal, middle, ventral and common meatuses, nasal septum, nasal gland, surrounding bony structures and fascial planes were free from any obvious abnormality.	In dorsal, sagittal and axial pre-contrast bone and soft tissue MPR, bilateral nasal turbinates, dorsal, middle, ventral and common meatuses, nasal septum, nasal gland, surrounding bony structures, choanae and fascial planes were free from any obvious abnormality.	In dorsal, sagittal and axial pre-contrast bone and soft tissue MPR, frontal and sphenoidal sinuses were clear. Congenitally septum of the sphenoidal sinuses was deviated towards left side, no other obvious abnormality was noticed in surrounding tissues	In dorsal, sagittal and axial pre-contrast bone and soft tissue MPR, no obvious abnormality was noticed in cervical tissues, cervical lymph nodes, cervical airways and cervical vertebrae.	In dorsal, sagittal and axial pre-contrast soft tissue and lungs MIP and MPR, pulmonary parenchyma, pulmonary vasculature, pleural and mediastinal spaces and mediastinal structures were free from any obvious abnormality.	In axial, dorsal and sagittal pre-contrast bone and soft tissue MPR, no obvious abnormality was noticed in extra-pulmonary structures. Mandibular, parotid, retropharyngeal, neck and thoracic lymph nodes including cranium and ear canals were free from any obvious abnormality.
Cat 04	In axial, dorsal and sagittal pre-contrast bone and soft tissue MPR, no obvious abnormality was noticed in nasal planum, nares, nasal conchae and nasal vestibule.	In dorsal, sagittal and axial pre-contrast bone and soft tissue MPR, bilateral sinus of dorsal conchae, nasal turbinates, dorsal, middle, ventral and common meatuses, nasal septum, nasal gland, surrounding bony structures and fascial planes were free from any obvious abnormality.	In dorsal, sagittal and axial pre-contrast bone and soft tissue MPR, bilateral nasal turbinates, dorsal, middle, ventral and common meatuses, nasal septum, nasal gland, surrounding bony structures, choanae and fascial planes were free from any obvious abnormality.	In dorsal, sagittal and axial pre-contrast bone and soft tissue MPR, frontal and sphenoidal sinuses were clear, no obvious abnormality was noticed in surrounding soft tissues.	In dorsal, sagittal and axial pre-contrast bone and soft tissue MPR, no obvious abnormality was noticed in cervical tissues, cervical lymph nodes, cervical airways and cervical vertebrae.	In dorsal, sagittal and axial pre-contrast soft tissue and lungs MIP and MPR, a mild crazy-paving pattern characterized by superimposition of the reticular pattern over ground glass opacification (GGO) was noticed in right cranial lung lobe. Rest of the pulmonary parenchyma, pulmonary vasculature, pleural and mediastinal spaces and mediastinal structures were free from any obvious abnormality	In axial, dorsal and sagittal pre-contrast bone and soft tissue MPR, no obvious abnormality was noticed in extra-pulmonary structures, mandibular, parotid, retropharyngeal, neck and thoracic lymph nodes and cranium. A bilateral horizontal ear canal was stenotic due to the irregular soft tissue attenuation adhering to the body tissue. Rest of the ear canal was free from any obvious abnormality.

Cat 05	In axial, dorsal and sagittal pre-contrast bone and soft tissue MPR, no obvious abnormality was noticed in nasal planum, nares and nasal vestibule.	In dorsal, sagittal and axial pre-contrast bone and soft tissue MPR, bilateral sinus of dorsal conchae, nasal turbinates, dorsal, middle, ventral and common meatuses, nasal septum, nasal gland, surrounding bony structures and fascial planes were free from any obvious abnormality.	In dorsal, sagittal and axial pre-contrast bone and soft tissue MPR, bilateral nasal turbinates, dorsal, middle, ventral and common meatuses, nasal septum, nasal gland, surrounding bony structures, choanae and fascial planes were free from any obvious abnormality.	In dorsal, sagittal and axial pre-contrast bone and soft tissue MPR, frontal and sphenoidal sinuses were clear. No obvious abnormality was noticed in surrounding soft tissues.	In dorsal, sagittal and axial pre-contrast bone and soft tissue MPR, no obvious abnormality was noticed in cervical tissues, cervical lymph nodes, cervical airways and cervical vertebrae.	In dorsal, sagittal and axial pre-contrast soft tissue and lungs MIP and MPR, a mild crazy-paving pattern superimposed over GGO, moderate peribronchovascular interstitial thickening, mild traction bronchiolectasis with architectural distortion and subjective thickening of the bronchial walls was noticed in caudal lobar of the left cranial, left caudal and right cranial lung lobes. A moderate mosaic pattern was also noticed in left and right caudal lung lobes. A prominent network of vasculature was perfusing pulmonary parenchyma. Cardiac silhouette was completely deviated towards right side due to congenital pectus excavatum. An irregular margination of prominent fat attenuation was noticed in the periphery of cardiac silhouette.	In axial, dorsal and sagittal pre-contrast bone and soft tissue MPR, no obvious abnormality was noticed in extra-pulmonary structures, mandibular, parotid, retropharyngeal, neck and thoracic lymph nodes and cranium. A bilateral horizontal ear canal was stenotic and bilateral tympanic bullae and tympanic cavities including musculotubal canals and cochlea were filled with hyper attenuating density. Septum bullae was still intact and tympanic membrane could not be appreciated. Vertical ear canal was free from any obvious abnormality.
Cat 06	In axial, dorsal and sagittal pre-contrast bone and soft tissue MPR, no obvious abnormality noticed in nasal planum, nares and nasal vestibule	In dorsal, sagittal and axial pre-contrast bone and soft tissue MPR, bilateral sinus of dorsal conchae, nasal gland, surrounding bony structures and fascial planes were free from any obvious abnormality. Overall thickening of the	In dorsal, sagittal and axial pre-contrast bone and soft tissue MPR, subjective thickening of the bilateral nasal turbinates was noticed. Dorsal, middle, ventral and common meatuses, nasal septum, nasal gland, surrounding bony structures, choanae	In dorsal, sagittal and axial pre-contrast bone and soft tissue MPR, frontal and sphenoidal sinuses were clear. No obvious abnormality was noticed in surrounding soft tissues	In dorsal, sagittal and axial pre-contrast bone and soft tissue MPR, no obvious abnormality was noticed in cervical tissues, cervical lymph nodes,	In dorsal, sagittal and axial pre-contrast soft tissue and lungs MIP and MPR, multiple end-on bronchi, and bronchioles were subjectively thickened in all lung lobes (mild), an irregular margination of fat attenuation noticed in the periphery of cardiac	In axial, dorsal and sagittal pre-contrast bone and soft tissue MPR, no obvious abnormality was noticed in extra-pulmonary structures, mandibular, parotid, retropharyngeal, neck and thoracic lymph nodes and cranium. A right

		nasal turbinates and narrowing of the meatuses with partial loss of the nasal septum was noticed in this region.	and fascial planes were free from any obvious abnormality.		cervical airways and cervical vertebrae.	silhouette. No other obvious abnormality was noticed in pulmonary parenchyma, thoracic vasculature, airways and mediastinal structures.	sided, unilateral, mildly stenotic horizontal ear canal was noticed with completely filled tympanic bullae along with tympanic cavity and musculotubal canal with hypo attenuating density compared to surrounding soft tissue density. Septum bullae was still intact.
Cat 07	In axial, dorsal and sagittal pre-contrast bone and soft tissue MPR, a moderate pathological necrosis of the dorsal and ventral conchae left nasal meatuses wider and more spacious as filtering passage.	In dorsal, sagittal and axial pre-contrast bone and soft tissue MPR, a moderate to severe unilateral filling of the right sinus of dorsal conchae with mixed attenuating density. Medial nasal gland and vomer were distorted in this region and septum was deviated towards right side. Surrounding bony structures and fascial planes were free from any obvious abnormality. A bilateral moderate to severe nasal turbinates lysis replaced by mixed attenuating density with complete loss of the ventral and common meatuses.	In dorsal, sagittal and axial pre-contrast bone and soft tissue MPR, a moderate to severe lysis of nasal turbinates on right side and moderate lysis noticed on left side, replaced by mixed attenuating density. Dorsal meatus was still clear and nasal septum was still intact. Nasal gland was distorted and surrounding bony structures including fascial planes were free from any obvious abnormality. Moderate filling of the choanae with mixed attenuating density.	In dorsal, sagittal and axial pre-contrast bone and soft tissue MPR, an isoattenuating density was filling right sphenoidal and frontal sinuses partially (mild).	In dorsal, sagittal and axial pre-contrast bone and soft tissue MPR, no obvious abnormality was noticed in cervical tissues, cervical lymph nodes, cervical airways and cervical vertebrae.	In dorsal, sagittal and axial pre-contrast soft tissue and lungs MIP and MPR, multiple end-on bronchi, and bronchioles were subjectively thickened in all lung lobes (mild). No other obvious abnormality noticed in pulmonary parenchyma, thoracic vasculature and airways. Mediastinal structures were free from any obvious abnormality	In axial, dorsal and sagittal pre-contrast bone and soft tissue MPR reverberating and refractory artifact was pronounced and masking minor details of the examined structures. No other obvious abnormality was noticed in extra-pulmonary structures, mandibular, parotid, retropharyngeal, neck and thoracic lymph nodes and cranium. A bilateral, mildly stenotic horizontal ear canals were noticed with completely filled tympanic bullae along with tympanic cavity and musculotubal canal with iso-attenuating density compared to surrounding soft tissue density. Septum bullae was still intact.

Cat 08	In axial, dorsal and sagittal pre-contrast bone and soft tissue MPR, an iso-attenuating density was occupying the right nasal opening partially (mild). A moderate pathological necrosis of the dorsal and ventral conchae left nasal meatuses wider and more spacious as filtering passage.	In dorsal, sagittal and axial pre-contrast bone and soft tissue MPR, a moderate to severe bilateral lysis of the turbinates replaced by an iso-attenuating density filling left sinus of dorsal conchae partially and almost complete entirety of the nasal cavity in this region except dorsal nasal meatuses. Medial nasal gland and vomer were distorted but septum was still intact. Surrounding bony structures and fascial planes were free from any obvious abnormality.	In dorsal, sagittal and axial pre-contrast bone and soft tissue MPR, a bilateral severe lysis of the turbinates was replaced by a partial (moderate) iso-attenuating density, filling the ventral aspect of the cavity predominantly ventral middle and common meatuses and choanae. Dorsal meatus was still clear and nasal septum was still intact. Nasal gland was distorted and surrounding bony structures including fascial planes were free from any obvious abnormality.	In dorsal, sagittal and axial pre-contrast bone and soft tissue MPR, bilateral mild to moderate filling of the frontal and sphenoidal sinuses with isoattenuating density compared to surrounding soft tissue.	In dorsal, sagittal and axial pre-contrast bone and soft tissue MPR, no obvious abnormality was noticed in cervical tissues, cervical lymph nodes, cervical airways and cervical vertebrae.	In dorsal, sagittal and axial pre-contrast soft tissue and lungs MIP and MPR, multiple end-on bronchi, and bronchioles were subjectively thickened in all lung lobes (mild). A multifocal mosaic pattern with mild peribronchovascular interstitial thickening were noticed in all lung lobes. No other obvious abnormality was noticed in pulmonary parenchyma, thoracic vasculature, airways and mediastinal structures.	In axial, dorsal and sagittal pre-contrast bone and soft tissue MPR, a bilateral, mildly stenotic horizontal ear canals were noticed with completely filled tympanic bullae along with tympanic cavity and musculotubal canal with iso-attenuating density compared to surrounding soft tissue density. Septum bullae was still intact. No other obvious abnormality was noticed in extra-pulmonary structures, mandibular, parotid, retropharyngeal, neck and thoracic lymph nodes and cranium.
Cat 09	In axial, dorsal and sagittal pre-contrast bone and soft tissue MPR, no obvious abnormality was noticed in nasal planum, nares, nasal conchae and nasal vestibule.	In dorsal, sagittal and axial pre-contrast bone and soft tissue MPR, bilateral sinus of dorsal conchae, nasal turbinates, dorsal, middle, ventral and common meatuses, nasal septum, nasal gland, surrounding bony structures and fascial planes were free from any obvious abnormality.	In dorsal, sagittal and axial pre-contrast bone and soft tissue MPR, bilateral nasal turbinates, dorsal, middle, ventral and common meatuses, nasal septum, nasal gland, surrounding bony structures, choanae and fascial planes were free from any obvious abnormality.	In dorsal, sagittal and axial pre-contrast bone and soft tissue MPR, frontal and sphenoidal sinuses were clear. No obvious abnormality was noticed in surrounding soft tissues.	In dorsal, sagittal and axial pre-contrast bone and soft tissue MPR, no obvious abnormality was noticed in cervical tissues, cervical lymph nodes, cervical airways and cervical vertebrae.	In dorsal, sagittal and axial pre-contrast soft tissue and lungs MIP and MPR, a clear but irregularly marginated GGO was noticed in left caudal lung lobe. No other obvious abnormality was noticed in pulmonary parenchyma, thoracic vasculature, airways and mediastinal structures.	In axial, dorsal and sagittal pre-contrast bone and soft tissue MPR, no obvious abnormality was noticed in extra-pulmonary structures. Mandibular, parotid, retropharyngeal, neck and thoracic lymph nodes including cranium and ear canals were free from any obvious abnormality.
Cat 10	In axial, dorsal and sagittal pre-contrast bone and soft tissue MPR, a moderate pathological	In dorsal, sagittal and axial pre-contrast bone and soft tissue MPR, a moderate to severe bilateral lysis of the turbinates replaced by	In dorsal, sagittal and axial pre-contrast bone and soft tissue MPR, a bilateral severe lysis of the turbinates was replaced by an iso-attenuating and air	In dorsal, sagittal and axial pre-contrast bone and soft tissue MPR, bilateral moderate filling of the frontal sinuses and bilateral complete filling	In dorsal, sagittal and axial pre-contrast bone and soft tissue MPR, no obvious abnormality was	In dorsal, sagittal and axial pre-contrast soft tissue and lungs MIP and MPR, multifocal mild to moderate size patches of the increased lung	In axial, dorsal and sagittal pre-contrast bone and soft tissue MPR, bilateral tympanic bullae along with tympanic cavity and musculotubal

	necrosis of the dorsal and ventral conchae left nasal meatuses wider and more spacious as filtering passage.	an iso-attenuating density mixed with air trapping regions filling bilateral sinus of dorsal conchae and almost complete entirety of the nasal cavity in this region. Medial nasal gland and vomer were distorted, and septum was lysed. Surrounding bony structures and fascial planes were free from any obvious abnormality.	trapping mixed density, filling the entirety of the nasal cavity, meatuses and choanae in this region. Nasal septum was thin but still intact. Nasal gland was completely distorted and surrounding bony structures including fascial planes were free from any obvious abnormality.	of the sphenoidal sinuses with iso-attenuating density compared to surrounding soft tissue.	noticed in cervical tissues, cervical lymph nodes, cervical airways and cervical vertebrae.	attenuation (GGO) with fading margins were noticed in left cranial and right middle lung lobes. No other obvious abnormality was noticed in pulmonary parenchyma, thoracic vasculature, airways and mediastinal structures.	canal were completely filled with iso-attenuating density compared to surrounding soft tissue density. Septum bullae was still intact. No other obvious abnormality was noticed in extra-pulmonary structures, mandibular, parotid, retropharyngeal, neck and thoracic lymph nodes and cranium.
Cat 11	In axial, dorsal and sagittal pre-contrast bone and soft tissue MPR, a moderate pathological necrosis of the dorsal and ventral conchae left nasal meatuses wider and more spacious as filtering passage in caudal nasal vestibule. Bilateral nares were stenotic and mildly blocked with isoattenuating density.	In dorsal, sagittal and axial pre-contrast bone and soft tissue MPR, a severe bilateral lysis of the turbinates replaced by an iso-attenuating density mixed with air trapping regions filling bilateral sinus of dorsal conchae and almost complete entirety of the nasal cavity in this region. Medial nasal gland and vomer were distorted. Septum was thin compare to normal but still intact. Surrounding bony structures and fascial planes were free from any obvious abnormality.	In dorsal, sagittal and axial pre-contrast bone and soft tissue MPR, a bilateral severe lysis of the turbinates was replaced by multifocal air trapped areas mixed with an iso-attenuating density filling the entirety of the nasal cavity, meatuses and choanae in this region. Nasal septum was thin but still intact. Nasal gland was completely distorted and surrounding bony structures including fascial planes were free from any obvious abnormality.	In dorsal, sagittal and axial pre-contrast bone and soft tissue MPR, bilateral iso-attenuating density compared to surrounding soft tissue was infiltrated mildly in frontal and sphenoidal sinuses.	In dorsal, sagittal and axial pre-contrast bone and soft tissue MPR, no obvious abnormality was noticed in cervical tissues, cervical lymph nodes, cervical airways and cervical vertebrae.	In dorsal, sagittal and axial pre-contrast soft tissue and lungs MIP and MPR, two focal areas of GGO with bronchiolectasis in distorted architecture were noticed in left cranial and caudal lung lobes. A generalized thickening of the first, second and third generation bronchial fields in all lung lobes. No other obvious abnormality was noticed in pulmonary parenchyma, thoracic vasculature, airways and mediastinal structures.	In axial, dorsal and sagittal pre-contrast bone and soft tissue MPR, bilateral tympanic bullae along with tympanic cavity and musculotubal canal were completely filled with iso-attenuating density compared to surrounding soft tissue density. Septum bullae was still intact. No other obvious abnormality was noticed in extra-pulmonary structures, mandibular, parotid, retropharyngeal, neck and thoracic lymph nodes and cranium.
Cat 12	In axial, dorsal and sagittal pre-contrast bone and soft tissue MPR,	In dorsal, sagittal and axial pre-contrast bone and soft tissue MPR, a severe bilateral lysis of	In dorsal, sagittal and axial pre-contrast bone and soft tissue MPR, a bilateral severe lysis of the	In dorsal, sagittal and axial pre-contrast bone and soft tissue MPR, right frontal and sphenoidal	In dorsal, sagittal and axial pre-contrast bone and soft tissue MPR, no	In dorsal, sagittal and axial pre-contrast soft tissue and lungs MIP and MPR, a severe diffuse hyper-	In axial, dorsal and sagittal pre-contrast bone and soft tissue MPR reverberating and

	right nasal passage was completely blocked while left nasal passage was stenotic and partially filled with isoattenuating density compared to surrounding tissue.	the turbinates was replaced by an iso-attenuating density filling (severe) bilateral sinus of dorsal conchae, complete entirety (severe) of the right nasal passage and almost complete entirety (severe) of the left nasal passage. Medial nasal gland was distorted, and septum was still intact. Surrounding bony structures and fascial planes were free from any obvious abnormality.	turbinates was partially (moderate) replaced by an iso-attenuating density compared to surrounding soft tissue in left nasal passage and completely replaced by a similar iso-attenuating density in right nasal passage. Nasal septum was still intact. Nasal gland was completely distorted and surrounding bony structures including fascial planes were free from any obvious abnormality.	sinuses were completely filled (severe) with iso-attenuating density compared to the surrounding soft tissue. A moderate to severe filling of the left frontal sinus was noticed with similar density. No other obvious abnormality was noticed in surrounding tissues.	obvious abnormality was noticed in cervical tissues, cervical lymph nodes, cervical airways and cervical vertebrae.	attenuating ground glass opacification was noticed in bilateral caudal and accessory lung lobes with multifocal areas of calcified bronchiolar walls. A single focal lesion with calcified bronchiolar wall and halo sign in surrounding parenchyma was noticed in left caudal lung lobe. Bilateral cranial and right middle lung lobes, mediastinal structures and pulmonary vasculature were free from any obvious abnormality.	refractory artifact was pronounced and masking minor details of the examined structures. Bilateral tympanic bullae along with tympanic cavity and musculotubal canal were completely filled with iso-attenuating density compared to surrounding soft tissue density. Septum bullae was still intact. No other obvious abnormality was noticed in extra-pulmonary structures, mandibular, parotid, retropharyngeal, neck and thoracic lymph nodes and cranium.
Cat 13	In axial, dorsal and sagittal pre-contrast bone and soft tissue MPR, no obvious abnormality was noticed in nasal planum, nares, nasal conchae and nasal vestibule.	In dorsal, sagittal and axial pre-contrast bone and soft tissue MPR, bilateral sinus of dorsal conchae, nasal turbinates, dorsal, middle, ventral and common meatuses, nasal septum, nasal gland, surrounding bony structures and fascial planes were free from any obvious abnormality.	In dorsal, sagittal and axial pre-contrast bone and soft tissue MPR, bilateral nasal turbinates, dorsal, middle, ventral and common meatuses, nasal septum, nasal gland, surrounding bony structures, choanae and fascial planes were free from any obvious abnormality.	In dorsal, sagittal and axial pre-contrast bone and soft tissue MPR, frontal and sphenoidal sinuses were clear. No obvious abnormality was noticed in surrounding tissues	In dorsal, sagittal and axial pre-contrast bone and soft tissue MPR, no obvious abnormality was noticed in cervical tissues, cervical lymph nodes, cervical airways and cervical vertebrae.	In dorsal, sagittal and axial pre-contrast soft tissue and lungs MIP and MPR, pulmonary parenchyma, pulmonary vasculature, pleural and mediastinal spaces and mediastinal structures were free from any obvious abnormality.	In axial, dorsal and sagittal pre-contrast bone and soft tissue MPR, no obvious abnormality was noticed in extra-pulmonary structures. Mandibular, parotid, retropharyngeal, neck and thoracic lymph nodes including cranium and ear canals were free from any obvious abnormality.
Cat 14	In axial, dorsal and sagittal pre-contrast bone and soft tissue MPR, an iso-attenuating density was occupying the area	In dorsal, sagittal and axial pre-contrast bone and soft tissue MPR, mild iso-attenuating density was occupying ventral aspect of the left nasal cavity with partial	In dorsal, sagittal and axial pre-contrast bone and soft tissue MPR, bilateral nasal turbinates, dorsal, middle, ventral and common meatuses, nasal septum, nasal gland, surrounding	In dorsal, sagittal and axial pre-contrast bone and soft tissue MPR, frontal and sphenoidal sinuses were clear. No obvious abnormality was	In dorsal, sagittal and axial pre-contrast bone and soft tissue MPR, no obvious abnormality was noticed in cervical	In dorsal, sagittal and axial pre-contrast soft tissue and lungs MIP and MPR, pulmonary parenchyma, pulmonary vasculature, pleural and mediastinal spaces and mediastinal	In axial, dorsal and sagittal pre-contrast bone and soft tissue MPR, no obvious abnormality was noticed in extra-pulmonary structures. Mandibular, parotid,

	of right ventral nasal concha, bone of ventral nasal concha and ventral nasal meatus at the level of canine teeth. Right canine tooth was fractured, body and nasal process of the incisive bone were not holding (104) canine properly due to the resorptive changes in tooth root.	(mild) loss of the turbinates. A continuity of the same tooth (104) related space occupying lesion was pushing maxillary turbinates dorsally and causing a mild lysis of the ventral turbinates. Sinus of dorsal conchae, dorsal and common meatuses, nasal septum, left-sided medial nasal gland, surrounding bony structures and fascial planes were free from any obvious abnormality.	bony structures, choanae and fascial planes were free from any obvious abnormality.	noticed in surrounding tissues	tissues, cervical lymph nodes, cervical airways and cervical vertebrae.	structures were free from any obvious abnormality.	retropharyngeal, neck and thoracic lymph nodes including cranium and ear canals were free from any obvious abnormality.
Cat 15	In axial, dorsal and sagittal pre-contrast bone and soft tissue MPR, right nasal opening was completely blocked with an iso-attenuating density while left nasal opening was stenotic. Overall stenosis of the nasal vestibule especially in right nasal passage was noticed due to the space occupying iso-attenuating density in nasal passage of this region.	In dorsal, sagittal and axial pre-contrast bone and soft tissue MPR, a severe bilateral lysis of the turbinates was replaced by an iso-attenuating density filling (severe) bilateral nasal passages completely and right sinus of dorsal concha partially. Medial nasal gland was distorted, dorsal meatus was clear, and septum was thin but still intact. Surrounding bony structures and fascial planes were free from any obvious abnormality.	In dorsal, sagittal and axial pre-contrast bone and soft tissue MPR, a bilateral moderate lysis of the turbinates was replaced by an iso-attenuating density compared to surrounding soft tissue Nasal septum was still intact. Nasal gland was completely distorted and surrounding bony structures including fascial planes were free from any obvious abnormality.	In dorsal, sagittal and axial pre-contrast bone and soft tissue MPR, bilateral frontal and sphenoidal sinuses were partially (mild) filled with an iso-attenuating density compared to the surrounding soft tissue. No other obvious abnormality was noticed in surrounding tissues.	In dorsal, sagittal and axial pre-contrast bone and soft tissue MPR, no obvious abnormality was noticed in cervical tissues, cervical lymph nodes, cervical airways and cervical vertebrae.	In dorsal, sagittal and axial pre-contrast soft tissue and lungs MIP and MPR, an overall increase in lung attenuation was noticed due to expiratory phase acquisition. A multifocal generalized thickening of the second and third-generation bronchioles was noticed in all lung fields. No other obvious abnormality was noticed in pulmonary parenchyma, pulmonary vasculature, pleural and mediastinal spaces and mediastinal structures.	In axial, dorsal and sagittal pre-contrast bone and soft tissue MPR, bilateral tympanic bullae along with tympanic cavity and musculotubal canal were moderately filled with iso-attenuating density compared to surrounding soft tissue density. Septum bullae was still intact. No other obvious abnormality was noticed in extra-pulmonary structures, mandibular, parotid, retropharyngeal, neck and thoracic lymph nodes and cranium.
Cat 16	In axial, dorsal and sagittal pre-	In dorsal, sagittal and axial pre-contrast bone	In dorsal, sagittal and axial pre-contrast bone and soft	In dorsal, sagittal and axial pre-contrast bone	In dorsal, sagittal and axial pre-	In dorsal, sagittal and axial pre-contrast soft tissue and	In axial, dorsal and sagittal pre-contrast bone

	contrast bone and soft tissue MPR, no obvious abnormality was noticed in nasal planum, nares, nasal conchae and nasal vestibule.	and soft tissue MPR, bilateral sinus of dorsal conchae, nasal turbinates, dorsal, middle, ventral and common meatuses, nasal septum, nasal gland, surrounding bony structures and fascial planes were free from any obvious abnormality.	tissue MPR, bilateral nasal turbinates, dorsal, middle, ventral and common meatuses, nasal septum, nasal gland, surrounding bony structures, choanae and fascial planes were free from any obvious abnormality.	and soft tissue MPR, frontal and sphenoidal sinuses were clear. No obvious abnormality was noticed in surrounding tissues	contrast bone and soft tissue MPR, no obvious abnormality was noticed in cervical tissues, cervical lymph nodes, cervical airways and cervical vertebrae.	lungs MIP and MPR, pulmonary parenchyma, pulmonary vasculature, pleural and mediastinal spaces and mediastinal structures were free from any obvious abnormality.	and soft tissue MPR, no obvious abnormality was noticed in extra-pulmonary structures. Mandibular, parotid, retropharyngeal, neck and thoracic lymph nodes including cranium and ear canals were free from any obvious abnormality.
Cat 17	In axial, dorsal and sagittal pre-contrast bone and soft tissue MPR, no obvious abnormality was noticed in nasal planum, nares, nasal conchae and nasal vestibule.	In dorsal, sagittal and axial pre-contrast bone and soft tissue MPR, bilateral sinus of dorsal conchae, nasal turbinates, dorsal, middle, ventral and common meatuses, nasal septum, nasal gland, surrounding bony structures and fascial planes were free from any obvious abnormality.	In dorsal, sagittal and axial pre-contrast bone and soft tissue MPR, bilateral nasal turbinates, dorsal, middle, ventral and common meatuses, nasal septum, nasal gland, surrounding bony structures, choanae and fascial planes were free from any obvious abnormality.	In dorsal, sagittal and axial pre-contrast bone and soft tissue MPR, frontal and sphenoidal sinuses were clear. No obvious abnormality was noticed in surrounding tissues	In dorsal, sagittal and axial pre-contrast bone and soft tissue MPR, megaesophagus was a conspicuous structure. No other obvious abnormality was noticed in cervical tissues, cervical lymph nodes, cervical airways and cervical vertebrae.	In dorsal, sagittal and axial pre-contrast soft tissue and lungs MIP and MPR, an overall increase in lung attenuation was noticed especially in the dependent regions of the lungs towards lying surface. A multifocal generalized thickening of the second and third-generation bronchioles was noticed in all lung fields. A conspicuous megaesophagus was pushing the trachea to right lateral and cardiac silhouette ventrally. A focal mild parabronchial interstitial thickening was noticed around second generation bronchi of bilateral caudal lung lobes.	In axial, dorsal and sagittal pre-contrast bone and soft tissue MPR, no obvious abnormality was noticed in extra-pulmonary structures. Mandibular, parotid, retropharyngeal, neck and thoracic lymph nodes including cranium and ear canals were free from any obvious abnormality.
Cat 18	In axial, dorsal and sagittal pre-contrast bone and soft tissue MPR, rostrally left nasal opening was	In dorsal, sagittal and axial pre-contrast bone and soft tissue MPR, a moderate bilateral lysis of the turbinates resulted in empty spaces	In dorsal, sagittal and axial pre-contrast bone and soft tissue MPR, a moderate bilateral lysis of the turbinates resulted in empty spaces due to the	In dorsal, sagittal and axial pre-contrast bone and soft tissue MPR, bilateral frontal sinuses were partially (mild) while sphenoidal sinuses	In dorsal, sagittal and axial pre-contrast bone and soft tissue MPR, no obvious abnormality was	In dorsal, sagittal and axial pre-contrast soft tissue and lungs MIP and MPR, right middle lung lobe was consolidated with air bronchograms visible in the	In axial, dorsal and sagittal pre-contrast bone and soft tissue MPR, bilateral tympanic bullae along with tympanic cavity and musculotubal

	completely blocked with an iso-attenuating density. Caudally, nasal vestibule was devoid of all conchae, leaving nasal vestibule wide and spacious. Surround tissues were free from any obvious abnormality.	due to the loss of the turbinates and remaining areas revealed an iso-attenuating density, coalescing with rest of the turbinates. Bilateral sinus of dorsal conchae was completely filled with an iso-attenuating density. Medial nasal gland was distorted, dorsal meatus was partially invaded with same density, and septum was thin but still intact. Surrounding bony structures and fascial planes were free from any obvious abnormality.	loss of the turbinates and remaining areas revealed an iso-attenuating density, coalescing with rest of the turbinates. Nasal septum was still intact. Nasal gland was completely distorted and surrounding bony structures including fascial planes were free from any obvious abnormality.	were completely (severe) filled with an iso-attenuating density compared to the surrounding soft tissue. No other obvious abnormality was noticed in surrounding tissues.	noticed in cervical tissues, cervical lymph nodes, cervical airways and cervical vertebrae.	region of architectural distortion. Parenchymal band of affected lung lobe was obvious. A focal mild parabronchial interstitial thickening was noticed around second generation bronchi of bilateral cranial lung lobes. No other obvious abnormality was noticed in pulmonary parenchyma, pulmonary vasculature, pleural and mediastinal spaces and mediastinal structures.	canal were moderately filled with iso-attenuating density compared to surrounding soft tissue density. Septum bullae was still intact. No other obvious abnormality was noticed in extra-pulmonary structures, mandibular, parotid, retropharyngeal lymph nodes and cranium.
Cat 19	In axial, dorsal and sagittal pre-contrast bone and soft tissue MPR, a severe stenosis of the nasal vestibule was noticed due to the coalescence of the thickened nasal conchae. Surrounding tissues were free from any obvious abnormality. Bilateral palatine fissure width was widened.	In dorsal, sagittal and axial pre-contrast bone and soft tissue MPR, bilateral sinus of dorsal conchae was clear. A severe thickening of the maxillary turbinates revealed an impression of mix attenuation masking medial nasal gland and nasal septum also. Dorsal, middle, common and ventral meatuses were completely disappeared due to turbinates thickening. Surrounding bony structures and fascial planes were free from any obvious abnormality.	In dorsal, sagittal and axial pre-contrast bone and soft tissue MPR, a generalized moderate bilateral thickening of the ethmoidal turbinates resulted in reduction of the meatuses and air filtering passages in this region. Nasal septum was still intact. Nasal gland could not be differentiated from coalesced turbinates density. Surrounding bony structures including fascial planes were free from any obvious abnormality.	In dorsal, sagittal and axial pre-contrast bone and soft tissue MPR, bilateral frontal sinuses were partially (mild) while sphenoidal sinuses were completely (severe) filled with an iso-attenuating density compared to the surrounding soft tissue. No other obvious abnormality was noticed in surrounding tissues.	In dorsal, sagittal and axial pre-contrast bone and soft tissue MPR, no obvious abnormality was noticed in cervical tissues, cervical lymph nodes, cervical airways and cervical vertebrae.	In dorsal, sagittal and axial pre-contrast soft tissue and lungs MIP and MPR, an overall increase in lung attenuation was noticed especially in the dependent region of the lungs towards lying surface. A multifocal generalized thickening of the second and third-generation bronchioles was noticed in all lung fields. No other obvious abnormality was noticed in pulmonary parenchyma, pulmonary vasculature, pleural and mediastinal spaces and mediastinal structures.	In axial, dorsal and sagittal pre-contrast bone and soft tissue MPR, multiple, distinct patches of the soft tissue attenuation attached to body tissue were blocking bilateral horizontal ear canals, indicating possible ceruminous wax. Bilateral tympanic bullae, septum bullae, tympanic cavity and musculotubal canals were free from any obvious abnormality. No other obvious abnormality was noticed in extra-pulmonary structures, mandibular, parotid, retropharyngeal, neck and thoracic lymph nodes and cranium.

Cat 20	In axial, dorsal and sagittal pre-contrast bone and soft tissue MPR, a bilateral severe stenosis of the nasal vestibule was noticed. A soft tissue attenuating density was filling the entirety of the right nasal passage while sever stenosis of the left nasal passage was noticed due to the coalescence of the thickened nasal conchae. Surround tissues were free from any obvious abnormality.	In dorsal, sagittal and axial pre-contrast bone and soft tissue MPR, bilateral sinus of dorsal conchae was clear. An iso-attenuating density was occupying the turbinates completely in right nasal passage and partially in left nasal passage of this region. Severe stenosis of the filtering passages was noticed in left nasal labyrinth. Dorsal, middle, common and ventral meatuses were completely filled with same abnormal density. Surrounding bony structures and fascial planes were free from any obvious abnormality.	In dorsal, sagittal and axial pre-contrast bone and soft tissue MPR, a generalized mild bilateral thickening of the ethmoidal turbinates resulted in reduction of the meatuses and air filtering passages of this region. Nasal septum and nasal glands were still intact. Mild invasion of the abnormal soft tissue attenuation was noticed in choanae also. Surrounding bony structures including fascial planes were free from any obvious abnormality.	In dorsal, sagittal and axial pre-contrast bone and soft tissue MPR, frontal and sphenoidal sinuses were clear. No obvious abnormality was noticed in surrounding tissues	In dorsal, sagittal and axial pre-contrast bone and soft tissue MPR, no obvious abnormality was noticed in cervical tissues, cervical lymph nodes, cervical airways and cervical vertebrae.	In dorsal, sagittal and axial pre-contrast soft tissue and lungs MIP and MPR, pulmonary parenchyma, pulmonary vasculature, pleural and mediastinal spaces and mediastinal structures were free from any obvious abnormality.	In axial, dorsal and sagittal pre-contrast bone and soft tissue MPR, bilateral tympanic bullae were completely filled while tympanic cavities were partially filled with an abnormal soft tissue attenuating density. No other obvious abnormality was noticed in extra-pulmonary structures, mandibular, parotid, retropharyngeal, neck and thoracic lymph nodes and cranium.
Cat 21	In axial, dorsal and sagittal pre-contrast bone and soft tissue MPR, a soft tissue attenuating density was filling the entirety of the bilateral nares and nasal vestibule. Surrounding tissues were free from any obvious abnormality. Bilateral palatine fissure width was widened.	In dorsal, sagittal and axial pre-contrast bone and soft tissue MPR, bilateral sinus of dorsal conchae was clear. Maxillary turbinates and meatuses were completely replaced by abnormal soft tissue attenuation. Nasal gland could not be differentiated from abnormal density although nasal septum was still intact. Surrounding bony structures and fascial	In dorsal, sagittal and axial pre-contrast bone and soft tissue MPR, an abnormal soft tissue attenuation was replacing the ethmoidal turbinates and filling the entirety of the bilateral nasal passages of this region. Nasal gland could not be differentiated from abnormal density although nasal septum was still intact. Surrounding bony structures and fascial planes were free from any obvious abnormality.	In dorsal, sagittal and axial pre-contrast bone and soft tissue MPR, frontal and sphenoidal sinuses were completely filled with an abnormal soft tissue attenuating density. A mild lysis of the bilateral cribriform plate was also noticed. No obvious abnormality was noticed in surrounding tissues.	In dorsal, sagittal and axial pre-contrast bone and soft tissue MPR, no obvious abnormality was noticed in cervical tissues, cervical lymph nodes, cervical airways and cervical vertebrae.	In dorsal, sagittal and axial pre-contrast soft tissue and lungs MIP and MPR, consolidation of the caudal lobar of the left cranial lung lobe was noticed with rough and irregular margins of the GGO and multiple mineral attenuating foci. Right cranial and middle lung lobes were also consolidated with similar concurrent patterns. Overall lung attenuation was increased. No other obvious abnormality was noticed in pulmonary	In axial, dorsal and sagittal pre-contrast bone and soft tissue MPR, no obvious abnormality was noticed in extra-pulmonary structures, retropharyngeal and parotid lymph nodes, cranium and ear canal. Mandibular lymph nodes were subjectively enlarged.

		planes were free from any obvious abnormality.				vasculature, pleural and mediastinal spaces and mediastinal structures.	
Cat 22	In axial, dorsal and sagittal pre-contrast bone and soft tissue MPR, a soft tissue attenuating density was occupying the nasal conchae causing and severe stenosis of the bilateral nasal vestibule. Surrounding tissues were free from any obvious abnormality.	In dorsal, sagittal and axial pre-contrast bone and soft tissue MPR, bilateral sinus of dorsal conchae, maxillary turbinates and meatuses were completely replaced by abnormal soft tissue attenuation. Nasal gland and nasal septum could not be differentiated from abnormally invading density. Surrounding bony structures and fascial planes were free from any obvious abnormality.	In dorsal, sagittal and axial pre-contrast bone and soft tissue MPR, an abnormal soft tissue attenuation was replacing the ethmoidal turbinates and filling the entirety of the bilateral nasal passages of this region. Nasal gland could not be differentiated from abnormal density although nasal septum was still intact. Surrounding bony structures and fascial planes were free from any obvious abnormality. Choanae was also filled by the same abnormal density of this region.	In dorsal, sagittal and axial pre-contrast bone and soft tissue MPR, left frontal sinus was moderately filled and bilateral sphenoidal sinuses were completely filled with an abnormal soft tissue attenuation. A mild lysis of the periorbital bony structures and bony nasal septum was noticed. No obvious abnormality was noticed in surrounding fascial planes.	In dorsal, sagittal and axial pre-contrast bone and soft tissue MPR, no obvious abnormality was noticed in cervical tissues, cervical lymph nodes, cervical airways and cervical vertebrae. Cervical esophagus was slightly gas filled.	In dorsal, sagittal and axial pre-contrast soft tissue and lungs MIP and MPR, a diaphragmatic outline was completely missing on left-sided thoracic cavity, allowing entire stomach and liver, major portion of the spleen and big tuft of intestines to penetrate in left thoracic cavity. Heart was pushed towards right side. Main stem bronchi and bronchioles were traceable in a small sized completely collapsed entire left lung field. Visible portion of the pulmonary parenchyma, pulmonary vasculature, and pleural space of right thoracic cavity were free from any obvious abnormality. Esophagus was slightly gas filled.	Animal was not positioned properly. Half of the right-sided lung was away from the acquisition beam. In axial, dorsal and sagittal pre-contrast bone and soft tissue MPR, bilateral tympanic bullae and tympanic cavities were severely filled with an abnormal soft tissue attenuation. No other obvious abnormality was noticed in extra-pulmonary structures, mandibular, parotid, retropharyngeal, neck and thoracic lymph nodes and cranium.

Table S2: Detailed radiographic findings of in 27 cats presented with acute/chronic infectious respiratory diseas signs

	Extra-thoracic abnormalities	Pleural space abnormalities	Pulmonary parenchyma	Mediastinum (cranial, medial and caudal)
Cat 01	Slightly ingesta filled stomach, liver silhouette beyond costochondral junction with prominent edge, no other significant extra-thoracic abnormality was noticed in L, R lateral and DV views	No significant pleural space abnormality was noticed in L, R lateral and DV views	A few prominent end-on bronchi were noticed in the middle and caudo-dorsal fields in right lateral view, rest of the pulmonary parenchyma, pulmonary vasculatures and airways were within normal range in L, R lateral and DV views	No significant mediastinal abnormality was noticed in L, R lateral and DV views
Cat 02	A significant volume of the gas was noticed in non-dependent areas of the stomach and visible sections of the small and large intestines in L, R lateral and DV views, a small 6.45 mm X 5.06 mm hyperplastic rounded opacity noticed at the distal bony aspect of the right 10th rib at the level of costochondral junction, rest of the extra-thoracic regions were without any obvious abnormality	No significant pleural space abnormality was noticed in L, R lateral and DV views	Pulmonary vasculatures and pulmonary parenchyma were within normal limits without any significant radiographic abnormality in L, R lateral and DV views	A significant volume of gas was noticed in thoracic esophagus with conspicuous delineation of the esophageal walls in both R and L lateral views, no other significant abnormalities were noticed in L, R lateral and DV views
Cat 03	Completely ingesta filled stomach was noticed, rest of the extra-thoracic regions were without any obvious abnormality in L, R lateral and DV views	No significant pleural space abnormality was noticed in L, R lateral and DV views	Mild bronchial pattern with thickening of the first-generation bronchi walls noticed in perihilar region, middle and caudo-dorsal lung fields in L, R lateral and DV views, rest of the pulmonary parenchyma and pulmonary vasculatures were within normal limit	A fat opacity noticed in cranial and middle mediastinum surrounding the cardiac silhouette as well in L and R lateral views, slight gas opacity noticed in esophagus in L, R lateral and DV views
Cat 04	Completely ingesta filled stomach was noticed, rest of the extra-thoracic regions were without any obvious abnormality in L, R lateral and DV views	No significant pleural space abnormality was noticed in L, R lateral and DV views	Pulmonary vasculatures and pulmonary parenchyma were within normal limits without any significant radiographic abnormality in L, R lateral and DV views	Multiple gas opacities noticed in cervical and thoracic part of the esophagus. An endotracheal tube was noticed in trachea placed till the level of cranial edge of second rib
Cat 05	A congenital malformation, pectus excavatum, characterized by dorsal deviation of the last three sternebrae was noticed in thoracic skeleton, resulting in an indentation of the caudoventral thoracic wall in L and R lateral views. A border effacement of the diaphragm with cardiac silhouette was also noticed in DV view	No significant pleural space abnormality was noticed in L, R lateral and DV views	A conspicuous vascular network of relatively normal size and range without any obvious abnormality in pulmonary parenchyma were noticed in L, R lateral and DV views	Cardiac silhouette was completely deviated towards R side and touching right thoracic wall in DV view, probably influenced by indentation and positioning. No other obvious abnormalities were noticed in cranial, middle, and caudal mediastinal spaces
Cat 06	Slightly gas filled stomach and transverse colon, rest of the extra-thoracic regions were without any obvious abnormality in L, R lateral and DV views	No significant pleural space abnormality was noticed in L, R lateral and DV views	Pulmonary vasculatures and pulmonary parenchyma were within normal limits without any significant radiographic abnormality in L, R lateral and DV views	Caudal vena cava was significantly smaller in diameter compared to the descending aorta with the CVC/Ao ratio of 0.73 (normal: 0.77 ± 0.02), possibly

				because of dehydration or hypovolemia. No other obvious abnormalities were noticed in cranial, middle, and caudal mediastinal spaces
Cat 07	Slightly gas filled stomach and transverse colon, liver silhouette was beyond the costochondral junction with sharp edges, rest of the extra-thoracic regions were without any obvious abnormality in L, R lateral and DV views	No significant pleural space abnormality was noticed in L, R lateral and DV views	Pulmonary vasculatures were within normal limit, mild to moderate multifocal bronchial pattern was noticed in all left and right lung lobes predominantly around peri-hilar region in L, R lateral and DV views	Caudal vena cava was significantly smaller in diameter compared to the descending aorta with the CVC/Ao ratio of 0.84, possibly because of dehydration or hypovolemia. No other obvious abnormalities were noticed in cranial, middle, and caudal mediastinal spaces
Cat 08	Slightly gas filled transverse colon, liver silhouette was beyond the costochondral junction with sharp edges, rest of the extra-thoracic regions were without any obvious abnormality in L, R lateral and DV views	No significant pleural space abnormality was noticed in L, R lateral and DV views	Subjectively right pulmonary artery and vein is thinner at the level of 9th rib compared to the left side in DV view, mild multifocal bronchial pattern was noticed in all left and right lung lobes predominantly in cranial and caudal lobar of the left lung lobe in L, R lateral and DV views	Caudal vena cava was significantly smaller in diameter compared to the descending aorta with the CVC/Ao ratio of 0.86, possibly because of dehydration or hypovolemia. No other obvious abnormalities were noticed in cranial, middle, and caudal mediastinal spaces
Cat 09	Ingesta and air bubbles were noticed in stomach, liver silhouette was slightly beyond the costochondral junction, no other obvious extra-thoracic abnormality was noticed in L, R lateral and DV views	No significant pleural space abnormality was noticed in L, R lateral and DV views	Mild bronchial pattern was noticed in right caudal lung lobe, rest of the pulmonary parenchyma and pulmonary vasculatures were within normal limit	No significant mediastinal abnormality was noticed in L, R lateral and DV views
Cat 10	A significant volume of the gas was noticed in non-dependent areas of the stomach and visible sections of the small intestine in L, R lateral and DV views, liver silhouette was slightly beyond the costochondral junction	No significant pleural space abnormality was noticed in L, R lateral and DV views	A multifocal mild bronchial pattern was noticed in left cranial and caudal lung lobes, rest of the pulmonary parenchyma and pulmonary vasculatures were within normal limit	No significant mediastinal abnormality was noticed in L, R lateral and DV views
Cat 11	A significant volume of the gas was noticed in non-dependent areas of the stomach and visible sections of the small and large intestines in L, R lateral and DV views, liver silhouette was slightly beyond the costochondral junction with sharp edges	No significant pleural space abnormality was noticed in L, R lateral and DV views	Pulmonary vasculatures were within normal limit, mild multifocal bronchial pattern was noticed in all left and right lung lobes predominantly in cranial and caudal lobes in L, R lateral and DV views	Caudal vena cava was significantly smaller in diameter compared to the descending aorta with the CVC/Ao ratio of 0.67, possibly because of dehydration or hypovolemia. No other obvious abnormalities were noticed in cranial, middle, and caudal mediastinal spaces
Cat 12	Moderate volume of gas was noticed in non-dependent areas of the stomach and visible sections of the small and large intestines in L, R lateral and DV views, no other obvious	No significant pleural space abnormality was noticed in L, R lateral and DV views	A mild unstructured interstitial framework was noticed generally in left and right-side lung lobes, quite prominent in caudal lobar of the left cranial and caudal lung lobes, pulmonary vasculatures were quite evident and their relative size was	A single small gassy region was noticed in thoracic esophagus just above the tracheal bifurcation in R lateral view, rest of the mediastinal spaces were within normal limit

	extra-thoracic abnormality was noticed in L, R lateral and DV views		within normal limits, multifocal mild bronchial pattern was also noticed in all left and right-sided lung lobes	
Cat 13	Slightly gas filled small intestines, no other obvious extra-thoracic abnormality was noticed in L, R lateral and DV views	No significant pleural space abnormality was noticed in L, R lateral and DV views	Pulmonary vasculatures and pulmonary parenchyma were within normal limits without any significant radiographic abnormality in L, R lateral and DV views	No significant mediastinal abnormality was noticed in L, R lateral and DV views
Cat 14	Slightly gas filled stomach, liver silhouette was beyond the costochondral junction with sharp edges in lateral views, no other obvious extra-thoracic abnormality was noticed in L, R lateral and DV views	No significant pleural space abnormality was noticed in L, R lateral and DV views	A mild bronchial pattern was noticed in dorso-caudal lung fields of the lateral radiographs, rest of the pulmonary vasculatures and pulmonary parenchyma were within normal limits without any significant radiographic abnormality in L, R lateral and DV views	No significant mediastinal abnormality was noticed in L, R lateral and DV views
Cat 15	Completely ingesta and gas bubbles filled stomach was noticed, liver silhouette was beyond the costochondral junction with sharp edges in lateral views, no other obvious extra-thoracic abnormality was noticed in L, R lateral and DV views	No significant pleural space abnormality was noticed in L, R lateral and DV views	A multifocal mild to moderate bronchial pattern was noticed in all left and right-side lung lobes, rest of the pulmonary vasculatures and pulmonary parenchyma were within normal limits without any significant radiographic abnormality in L, R lateral and DV views	A prominent fat opacity noticed around apex of the cardiac silhouette in lateral views, rest of the mediastinal spaces were within normal limit
Cat 16	No significant extra-thoracic abnormality was noticed in L, R lateral and DV views	No significant pleural space abnormality was noticed in L, R lateral and DV views	A conspicuous thin vascular network of relatively normal size and range without any obvious abnormality in pulmonary parenchyma were noticed in L, R lateral and DV views	Caudal vena cava was significantly smaller in diameter compared to the descending aorta with the CVC/Ao ratio of 0.74, possibly because of dehydration or hypovolemia. No other obvious abnormalities were noticed in cranial, middle, and caudal mediastinal spaces
Cat 17	Highly gas filled stomach, liver silhouette beyond costochondral junction, no other obvious extra-thoracic abnormality was noticed in L, R lateral and DV views	No significant pleural space abnormality was noticed in L, R lateral and DV views	Pulmonary vasculatures and pulmonary parenchyma were within normal limits without any significant radiographic abnormality in L, R lateral and DV views	No significant mediastinal abnormality was noticed in L, R lateral and DV views
Cat 18	Completely ingesta filled stomach, liver silhouette beyond costochondral junction, no other obvious extra-thoracic abnormality was noticed in L, R lateral and DV views	No significant pleural space abnormality was noticed in L, R lateral and DV views	Pulmonary vasculatures were within normal limit, a severe alveolar pattern of the collapsed right middle lung lobe was noticed with lobar sign, air bronchogram and border effacement of the cardiac silhouette touching affected lung lobe in R lateral and DV views, a generalized unstructured mild interstitial and mild bronchial pattern was also noticed in left caudal lung lobe in DV view	No significant mediastinal abnormality was noticed in L, R lateral and DV views

Cat 19	Completely ingesta filled stomach, liver silhouette beyond costochondral junction, no other obvious extra-thoracic abnormality was noticed in L, R lateral and DV views	No significant pleural space abnormality was noticed in L, R lateral and DV views	Pulmonary vasculatures were within normal limit, a generalized unstructured mild interstitial pattern was noticed in all lung lobes in L, R lateral and DV view	In middle mediastinum, a fat opacity was causing border effacement of the cardiac silhouette with sternal border of the thoracic cavity in L and R lateral views, no other obvious abnormalities were noticed in cranial, middle, and caudal mediastinal spaces
Cat 20	Moderately gas filled stomach, an endotracheal tube was noticed in trachea, no other obvious extra-thoracic abnormality was noticed in L, R lateral and DV views	No significant pleural space abnormality was noticed in L, R lateral and DV views	Pulmonary vasculatures and pulmonary parenchyma were within normal limits without any significant radiographic abnormality in L, R lateral and DV views	No significant mediastinal abnormality was noticed in L, R lateral and DV views
Cat 21	Moderately ingesta filled stomach, gas filled visible sections of the small and large intestines, an endotracheal tube was noticed in trachea, liver silhouette beyond the costochondral junction	No significant pleural space abnormality was noticed in L, R lateral and DV views	Pulmonary vasculatures were within normal limit. Right cranial, middle lung lobes and caudal lobar of the left cranial lung lobe were collapsed with distinct silhouette sign in L, R lateral and DV views, border effacement of the cardiac silhouette with collapsed lung lobes was noticed in DV view, air bronchograms within the affected lung lobes and mild bronchial patterns of left side lung lobes were noticed in DV view	No significant mediastinal abnormality was noticed in L, R lateral and DV views
Cat 22	A major portion of the abdominal organs like stomach, whole small intestine, liver, ascending, transverse and half of the descending colon and head of the spleen was noticed in thoracic cavity in both L, R lateral and DV views, a major herniation noticed on left side of the thoracic cavity	Not visible in L, R lateral and DV views because of the abdominal content's herniation	An inconspicuous right cranial lung lobe was noticed in DV view. An indistinct, small but semi-intact portion of the diaphragm effacing the right-sided cranially migrated hepatic silhouette was noticed in right thoracic cavity in DV view, pulmonary parenchyma could not be appreciated in L, R lateral views, trachea was elevated and touching the dorsal border of the thoracic cavity	Not visible in L, R lateral and DV views because of the abdominal content's herniation
Cat 23	Poor serosal details for abdominal organs probably because of the very young age or slight fluid in the abdominal cavity, liver silhouette was quite enlarged and beyond the costochondral junction in lateral views, no other obvious extra-thoracic abnormality was noticed in L, R lateral and VD views	Gravity dependent fluid opacity was covering more than half of the thoracic cavity in L and R views	Severe alveolar pattern with air bronchograms noticed in all lung lobes in VD view. Trachea was slightly elevated, and a reduced tracheal-spinal angle was noticed in L and R lateral views. Pulmonary parenchyma was obscured by superimposed fluid opacity in VD view. A significant rounding and retraction of all lung lobe's margins were noticed in VD view, Diaphragmatic outline was obscured completely in VD view and more than half in lateral views.	Cardiac silhouette was completely obscured by fluid opacity. Cranial, middle and caudal mediastinal spaces could not be traced because of the massive fluid opacity in chest cavity, Cranial mediastinal retrosternal lymph node enlargement
Cat 24	Serosal details were lost for abdominal organs probably because of the very young	Pleural space was completely filled with fluid opacity except around	Severe alveolar pattern with air bronchograms noticed in all lung lobes in VD view. Only left and	Cardiac silhouette was completely obscured by fluid opacity. Cranial, middle

	age or fluid in abdominal cavity, liver silhouette could be traced until mid-ventral abdomen, no other obvious extra-thoracic abnormality was noticed in L, R lateral and VD views	caudal left and right lobes in L, R lateral and VD views	right caudal lung lobes were visible. A well-rounded cavitated lesion was noticed in R lateral view in right caudal lung lobe. Trachea was elevated in R and L lateral views and touching the dorsal border of the thoracic cavity. Scalloping of the diaphragm with rounding of the caudal margins of the caudal lung lobes were noticed in R lateral and VD view	and caudal mediastinal spaces could not be traced because of the massive fluid opacity in chest cavity
Cat 25	Poor serosal details for abdominal organs probably because of the very young age or slight fluid in the abdominal cavity, liver silhouette could be traced until mid-ventral abdomen, no other obvious extra-thoracic abnormality was noticed in L, R lateral and VD views	Gravity dependent fluid opacity was covering half of the thoracic cavity in L and R views	Severe alveolar pattern with air bronchograms noticed in all lung lobes in VD view. Only cranial lobar of the left cranial and left and right caudal lung lobes were visible in L and R lateral views. Trachea was slightly elevated, and a reduced tracheal-spinal angle was noticed in L and R lateral views. Pulmonary parenchyma was obscured by superimposed fluid opacity in VD view. A significant rounding and retraction of left cranial and left and right caudal lung lobes margins were noticed in VD view	Cardiac silhouette was completely obscured by fluid opacity. Cranial, middle and caudal mediastinal spaces could not be traced because of the massive fluid opacity in chest cavity, Cranial mediastinal retrosternal lymph node enlargement
Cat 26	Slightly gas filled stomach, liver silhouette was quite enlarged and beyond the costochondral junction in lateral views, no other obvious extra-thoracic abnormality was noticed in L, R lateral and VD views	Gravity dependent fluid opacity was covering more than half of the thoracic cavity in L and R views	Severe alveolar pattern with collapsed right cranial and middle; and left cranial lung lobes manifesting silhouette sign in L and R lateral views. Air bronchograms noticed in right middle and left cranial lung lobes in VD view. Trachea was slightly elevated, and a reduced tracheal-spinal angle was noticed in L and R lateral views. Pulmonary parenchyma was obscured by superimposed fluid opacity in VD view. A significant rounding and retraction of all lung lobe's margins were noticed in VD view, Diaphragmatic outline was obscured completely in VD view and more than half in lateral views.	Cardiac silhouette was completely obscured by fluid opacity. Cranial, middle and caudal mediastinal spaces could not be traced because of the massive fluid opacity in chest cavity. A double opacity was suspected at the level of retrosternal lymph nodes.
Cat 27	Slightly gas and ingesta filled stomach, liver silhouette was quite enlarged and beyond the costochondral junction in lateral views, no other obvious extra-thoracic abnormality was noticed in L, R lateral and VD views	Gravity dependent fluid opacity was covering more than half of the thoracic cavity in L and R views	Severe alveolar pattern with collapsed right cranial and middle; and left cranial lung lobes. An obvious air-filled pocket (pneumatocoele) noticed at the level of right cranial lung lobe in VD view. Air bronchograms noticed in right middle and left and right cranial lung lobes in lateral and VD views. Trachea was elevated, and a reduced tracheal-spinal angle was noticed in L and R lateral	Cardiac silhouette was completely obscured by fluid opacity. Cranial, middle and caudal mediastinal spaces could not be traced because of the massive fluid opacity in chest cavity. A double opacity was suspected at the level of retrosternal lymph nodes.

			views. Whole pulmonary parenchyma except pneumatocoele was obscured by superimposed fluid opacity in VD view. A significant rounding and retraction of all lung lobe’s margins were noticed in VD view, Diaphragmatic outline was completely obscured in VD and lateral views.	
